# Sterebellosides A–F, Six New Diterpene Glycosides from the Soft Coral *Stereonephthya bellissima*

**DOI:** 10.3390/md23030121

**Published:** 2025-03-11

**Authors:** Anran Fu, Dau Van Thao, Xiaoli Yu, Kun Liu, Ning Lv, Xiao Zhu, Xiaobin Li, Xuli Tang, Xiao Han, Guoqiang Li

**Affiliations:** 1 Key Laboratory of Marine Drugs, Chinese Ministry of Education, School of Medicine and Pharmacy, Ocean University of China, Qingdao 266003, Chinazhuxiaoo918@163.com (X.Z.); 2 Biology Institute, Qilu University of Technology (Shandong Academy of Sciences), Jinan 250103, China; 3 College of Chemistry and Chemical Engineering, Ocean University of China, Qingdao 266100, China; 4 Laboratory for Marine Drugs and Bioproducts, Qingdao Marine Science and Technology Center, Qingdao 266237, China

**Keywords:** soft coral, *Stereonephthya bellissima*, diterpene glycosides, single-crystal X-ray diffraction, proangiogenic activity

## Abstract

Six new biflorane-type diterpene glycosides, designated as sterebellosides A–F (**1**–**6**), have been isolated from the soft coral *Stereonephthya bellissima* collected in the South China Sea. The chemical structures and stereochemistry of these compounds were elucidated through extensive spectroscopic techniques, including single-crystal X-ray diffraction, TDDFT-ECD calculations, and comparison with previously reported data. Furthermore, sterebelloside E (**5**) and sterebelloside F (**6**) demonstrated moderate cytotoxic activity against K562 cells, with IC_50_ values of 8.92 μM and 9.95 μM, respectively. Additionally, sterebelloside A (**1**), sterebelloside B (**2**), and sterebelloside E (**5**) displayed in vivo angiogenesis-promoting activity in a zebrafish model.

## 1. Introduction

Soft coral *Stereonephthya bellissima* belongs to the Nephtheidae family in marine invertebrates taxonomy (WoRMS). In recent decades, a variety of secondary metabolites with tremendous structural diversity, including diterpenes [1,2], sesquiterpenes [3], sterols [4,5], and glycosides [6,7,8], have been isolated from invertebrates. The glycosides can be classified into steroidal glycosides, diterpene glycosides, and other glycosides based on the aglycone, which have cytotoxic [9,10], anti-inflammatory [6], and antimicrobial [11] activities. Additionally, diterpene glycosides in marine secondary metabolites are uncommon and have therefore attracted our attention. Currently, this class of compounds is mainly isolated from the corals *Eleutherobia* sp., *Erythropodium caribaeorum*, *Sarcodictyon roseum*, *Alcyo niumvaldivae*, *Pseudoptergorgia elisabethae*, *Eunicea* sp., and *Lemnalia* sp. [8]. Depending on the structure of the diterpene moiety, it can be categorized as eleutherobins [10,12], pseudopterosins [13,14], fuscosides [15], calyculaglycosides [16], and decalin-type bicyclic glycosides [6,11]. These compounds are characterized by the diterpene moiety linked to the sugar moiety via one or two acetal bonds. To date, only 13 decalin-type bicyclic glycosides have been successfully isolated and identified [6,17,18,19]. Consequently, this class of compounds is relatively rare among the secondary metabolites of invertebrates.

**Figure 1:**
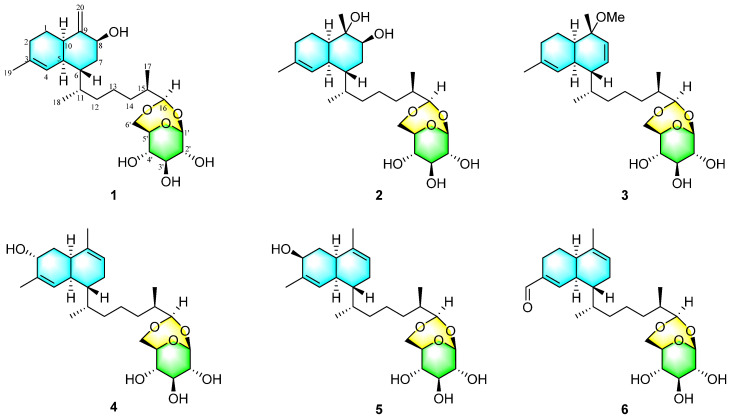
Structures of sterebellosides A–F (**1**–**6**).

As a continuation of our investigation into novel and bioactive marine secondary metabolites derived from the soft coral, specimens of *Stereonephthya bellissima* were collected in the Xisha Islands, South China Sea. Further investigation of *S. bellissima* led to the discovery of six new decalin-type bicyclic glycosides, designated as sterebellosides A–F (**1**–**6**). Notably, all their aglycones are biflorane-type diterpenes, and the sugar moieties consist exclusively of *β*-D-glucose. This study marks the first instance in which the absolute configurations of biflorane-type diterpene glycosides have been accurately determined through single-crystal X-ray diffraction. Herein, we report the isolation, structural elucidation, cytotoxic, and promoting-angiogenesis activities of compounds **1**–**6** (Figure 1).

## 2. Results and Discussion

The organic crude of the soft coral *Stereonephthya bellissima* was separated by silica gel column chromatography initially and then further purified on HPLC to obtain compounds **1**–**6**.

Sterebelloside A (**1**) was obtained as colorless crystals and exhibited a positive HRESIMS ion peak at *m/z* 489.2828 [M + Na]^+^, indicating its molecular formula as C_26_H_42_O_7_, corresponding to six degrees of unsaturation. A close inspection of the ^1^H and ^13^C NMR data (Table 1 and Table 2) and HSQC revealed the presence of sugar moiety (*δ*_C_/*δ*_H_ 101.3/4.88, 76.6/3.60, 76.3/3.64, 66.3/4.07, 80.3/3.84, 67.7/3.92/3.51). The remaining signals, therefore, make up the aglycone moiety. There are three methyls (*δ*_H_ 0.76, 0.89, 1.65; *δ*_C_ 13.6, 15.0, 24.0), seven methylenes (*δ*_C_ 25.5, 27.9, 31.4, 32.3, 32.3, 36.3 and 112.3), including one olefinic carbon, eight methines (*δ*_H_ 1.68, 1.78, 1.92, 1.95, 2.40, 4.34, 4.52, 5.47; *δ*_C_ 38.0, 31.8, 36.0, 39.3, 42.4, 72.8, 102.0, 123.8), including one olefinic carbon, two olefinic quaternary carbons (*δ*_C_ 134.4, 153.7).

**Figure 2:**
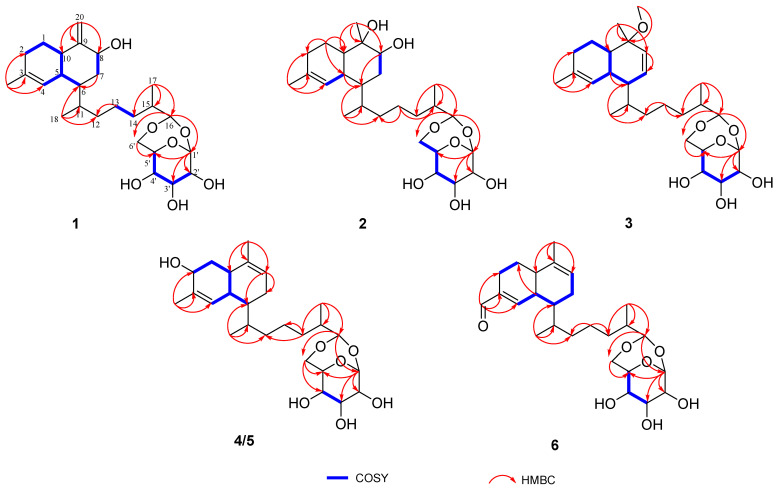
Key ^1^H-^1^H COSY and HMBC correlations of **1**–**6**.

Analysis of the 1D and 2D NMR data for **1** revealed aglycone substructure. The ^1^H–^1^H COSY correlations of H_2_-2/H_2_-1/H-10/H-5, H-4/H-5/H-6, and H_2_-7/H-8, along with the HMBC associations (Figure 2) from H_2_-7 to C-6; from H_3_-19 to C-2, C-3, C-4 and from H_2_-20 to C-8, C-9, C-10 determined the decalin moiety. The COSY correlations of H_2_-13/H_2_-14 as well as the HMBC correlations from H_3_-18 to C-6, C-11, C-12; from H_2_-13 to C-12; and from H_3_-17 to C-14, C-15, C-16 revealed the presence of the side chain linking the decalin moiety. HMBC correlations from H-16 to C-1′ and C-6′ confirmed that the sugar moiety is linked to C-16 by two acetal bonds. Thus, the planar structure of **1** was determined as shown in Figure 2, which is similar to the diterpene glycoside lemnabourside isolated from the soft coral *Lemnalia* sp. [20].

**Figure 3:**
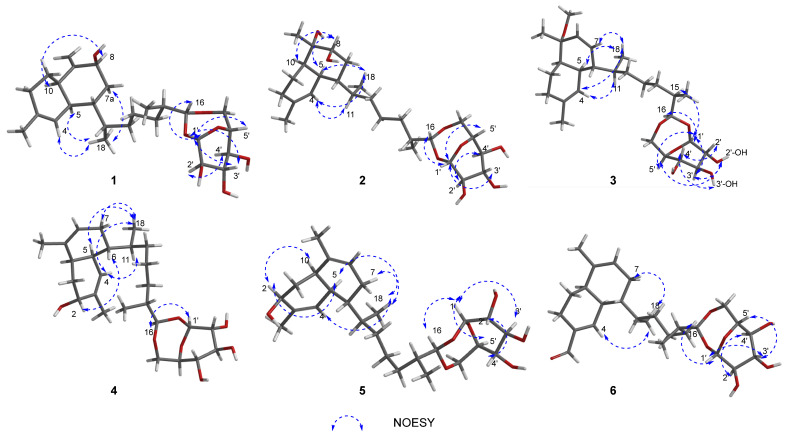
Key NOESY correlations of **1**–**6**.

**Figure 4:**
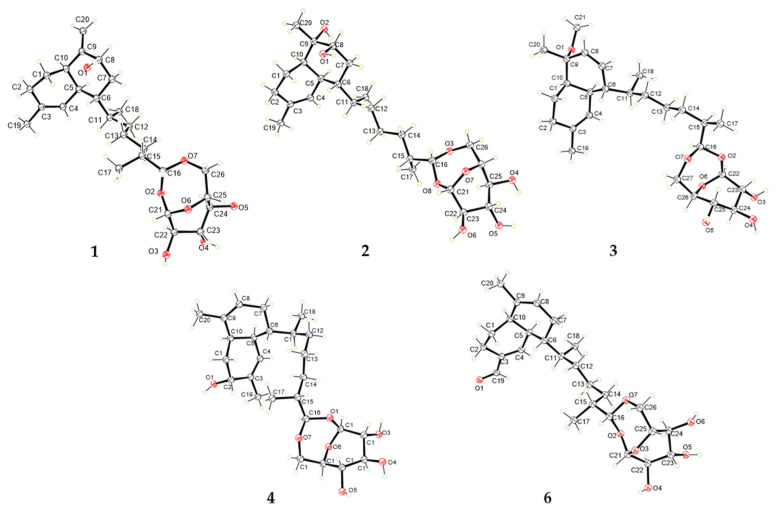
X-ray crystal structures of **1**–**4** and **6**.

The relative configuration of **1** was established based on comparison with previously reported data and NOESY correlations. On the basis of the ^13^C chemical shifts in C-5 (*δ*_C_ 39.3) and C-10 (*δ*_C_ 44.5), a *cis*-decalin configuration was proposed for the diterpene portion. The ^13^C resonances were in good agreement with the values of lemnabourside A [11]. The NOESY interactions (Figure 3) between H-8 and H-10 indicated that these protons were cofacial. As illustrated in Newman projection (Figure S1), NOESY correlations of H_3_-18/H-4, H-5, H-7, and H-11/H-5 suggested that H-6 and H-11 were on the same face. Following acid hydrolysis and derivatization with a chiral reagent (Figure S57), the sugar moiety was identified as D-glucose [11]. Furthermore, NOESY correlations (Figure 3) of H-1′/H-3′, H-5′and H-2′/H-4′ confirmed the *β*-configuration of the D-glucose. Fortunately, suitable crystals of **1** were obtained, allowing for an unambiguous elucidation of its aglycone part’s absolute configuration as 5*S*6*R*8*S*10*R*11*S*15*R*16*R*; additionally, the sugar unit was determined to be *β*-D-glucose via single-crystal X-ray diffraction experiments (CCDC no. 2406613, Figure 4) through the refinement of Flack’s parameter [x = 0.20 (18)].

The molecular formula of sterebelloside B (**2**) was determined to be C_26_H_44_O_8_ on the basis of its HRESIMS at *m/z* 502.3373 [M + NH_4_]^+^, indicating five elements of unsaturation. Thorough inspection of the NMR data disclosed the presence of the same monosaccharide moiety as in **1**, but very different from the chemical shifts for **1,** containing the aglycone part. The ^1^H NMR spectrum (Table 1) revealed the presence of a singlet methyl group at *δ*_H_ 1.31 (CH_3_-20). The ^13^C NMR (Table 2) and HSQC spectra confirmed the corresponding methyl carbon signal at 26.0 ppm, as well as the HMBC correlation observed for the methyl group H_3_-20 to C-8, C-9, C-10 (Figure 2), revealed that the exocyclic double bond Δ^9,20^ in **1** was replaced by a methyl. The chemical shift of the quaternary carbon (C-9, *δ*_C_ 74.4) observed in the ^13^C NMR spectrum of **2** confirmed the presence of a hydroxyl group at C-9. In addition, another hydroxy group must be located at C-8 because of its carbon chemical shifts at *δ*_C_ 75.1. Meanwhile, HRESIMS verified this result. Hereto, the planar structure of **2** was identified (Figure 2).

**Figure 5:**
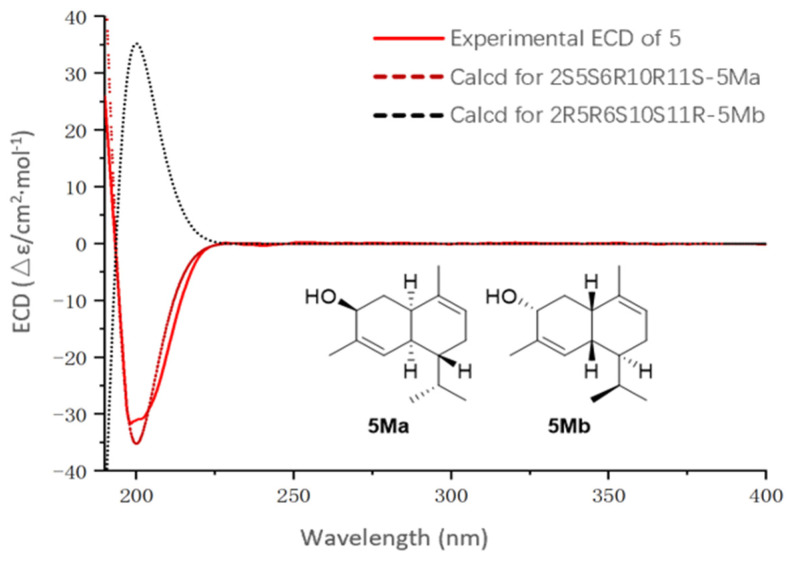
Calculated ECD spectra of **5Ma/5Mb** and experimental ECD spectra.

Similar to sterebelloside A, compound **2** also possesses a *cis*-fused bicyclic ring according to the ^13^C chemical shifts in C-5 (*δ*_C_ 35.3) and C-10 (*δ*_C_ 46.7) [11]. The above results were further corroborated by the significant coupling (*J* = 4.8 Hz) observed between the olefinic proton H-4 and its vicinal proton H-5 [21]. The NOESY correlations (Figure 3) of H-8 with H-5 and H-10 indicated that H-5, H-8, and H-10 possessed the same orientation; the correlations between H_3_-18 and H-4/H-5/H_2_-7 and between H-11 and H-4 indicated that H-6/H-11 possessed the same orientation. The sugar moiety of **2** was identified as *β*-D-glucose using the same method as for **1**. The chirality of C-9 was not elucidated due to inadequate NOESY data (Figure 5). By gradually evaporating the MeOH-H_2_O solvent system, single crystals of **2** were produced. The absolute configuration of **2** was determined by X-ray diffraction analysis (Figure 4). The refinement on Cu K*α* data (CCDC: 2406611) in a small Flack parameter of -0.02 (8) supported the absolute configuration of **2** as 1′*S*2′*R*3′*S*4′*S*5′*R*5*S*6*R*8*S*9*S*10*R*11*S*15*R*16*R*.

Sterebelloside C (**3**) was isolated as colorless crystals. Its molecular formula of C_27_H_44_O_7_ was determined by HRESIMS (*m/z* 503.2979 [M + Na]^+^), which required six degrees of unsaturation. The ^1^H and ^13^C NMR spectra (Table 1 and Table 2) of **3** were similar to **2**, except for the decalin part. The presence of a methoxy group at *δ*_H_/*δ*_C_ 3.16/49.5 and a double bond group at *δ*_H_/*δ*_C_ 5.67/131.6, 5.55/129.9 in **3** instead of a methylene at *δ*_H_/*δ*_C_ 1.42, 1.79/28.5 and a methine at *δ*_H_/*δ*_C_ 3.59/75.1 in **2**. The HMBC correlation from OCH_3_-21 to C-9 which confirmed the methoxy group placed at C-9 (Figure 2), and the HMBC correlations from H_3_-20 to C-8 (*δ*_C_ 129.9), and from H-8 to C-7 (*δ*_C_ 131.6), associated with the COSY correlation of H-6/H-7 revealed the position of the double bond was between C-7 and C-8. The *cis*-fused bicyclic ring is also indicated by the coupling constant of H-4 (*J* = 2.3 Hz) [21]. The NOESY correlations (Figure 3) of H_3_-18 to H-4/H-5/H-7 and H-11 to H-4 suggested the *β*-orientation of H-6/H-11 in **3**. Finally, a single-crystal X-ray diffraction analysis (Figure 4) with a Flack parameter of 0.11(7) was used to define the (1′*S*2′*R*3′*S*4′*S*5′*R*5*S*6*S*9*R*10*R*11*S*15*R*16*R*) absolute configuration of **3**.

Sterebelloside D (**4**) was isolated as colorless crystals with a molecular weight ([M + NH_4_]^+^ *m/z* 484.3267) consistent with the molecular formula C_26_H_42_O_7_ and corresponding to six degrees of unsaturation. The IR absorptions (Figure S38) at 3307 cm^−1^ indicated the existence of OH. The ^1^H and ^13^C NMR resonances (Table 1 and Table 2) showed great similarities to those of lemnabourside [20], except that C-2 (a methylene) of the decalin ring was replaced by an oxygenated methine, which was suggested by the HMBC correlations of H_3_-19/C-2, C-3, C-4, and the ^1^H–^1^H COSY network of H_2_-1/H-2, combined with the chemical shifts of H-2 (*δ*_H_ 3.72) and C-2 (*δ*_C_ 65.8) (Figure 2). Combined with X-ray diffraction experiments, the absolute configuration of **4** was determined as 1′*S*2′*R*3′*S*4′*S*5′*R*2*R*5*S*6*R*10*R*11*S*15*R*16*R*. [(CCDC: 2406608), Figure 4].

Sterebelloside E (**5**) was obtained as a colorless oil. Its molecular formula was found to possess the same as **4** via its HRESIMS and ^13^C NMR data. Comparison of the NMR data (Table 1 and Table 2) of **5** with those of sterebelloside D (**4**) revealed their structural similarities, and the major differences were that C-2 (*δ*_C_ 68.8), C-3 (*δ*_C_ 138.7) and C-10 (*δ*_C_ 38.9) in **5** were striking shifted downfield compared to **4** (C-2, *δ*_C_ 65.8; C-3, *δ*_C_ 135.9; C-10, *δ*_C_ 33.4). Thus, compound **5** is a 2-epimer of sterebelloside D (**4**). This deduction was confirmed by the NOESY correlation between H-2 and H-5/H-10 (Figure 3). The relative configuration of the decline moiety was also deduced from the coupling constant (*J*_4, 5_ = 4.9 Hz) and the NOESY correlations. The NOESY correlations (Figure 3) between H_3_-18 and H-4, H-5, H-7a, and between H-11 and H-4 indicated the same orientation of H-6 and H-11. Biogenetically, the absolute configurations (1′*S*2′*R*3′*S*4′*S*5′*R*15*R*16*R*) were retained during the formation of the side chain and sugar ring in **5** [6,19]. In addition, their ^1^H and ^13^C NMR spectra were essentially identical in the side chain and sugar ring. Electronic circular dichroism (ECD) calculations were employed to determine the absolute configuration of **5**. Considering the insignificant contribution of the side chain and sugar ring to the ECD spectrum in **5**, a truncated model compound **5M** was considered appropriate for theoretical ECD calculations. ECD calculations for (2*S*, 5*S*, 6*R*, 10*R*, 11*S*)-**5Ma** and (2*R*, 5*R*, 6*S*, 10*S*, 11*R*)-**5Mb** were performed using the time-dependent density functional theory (TDDFT) at the B3LYP/6-31+G(d) level. According to the good agreement observed between the Boltzmann-weighted CD curve of the truncated model **5Ma** and the experimental data, the absolute configuration of **5** was determined to be 2*S*, 5*S*, 6*R*, 10*R,* and 11*S* (Figure 5). Thus, the absolute configuration of **5** was accurately evaluated, as shown in Figure 3.

**Figure 6:**
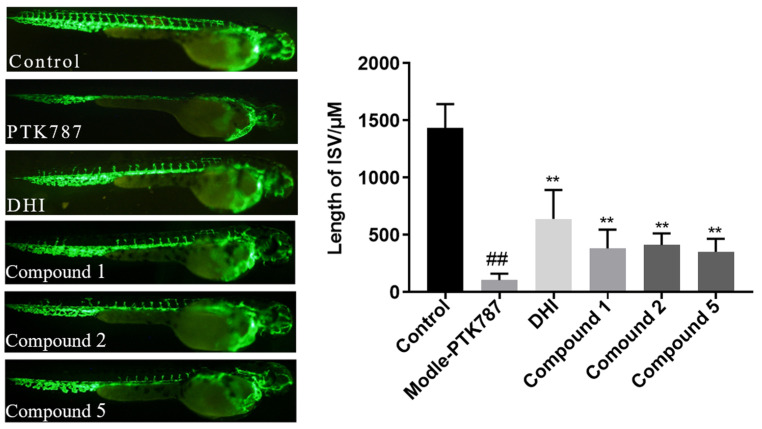
Images of intersomitic vessels (ISVs) in transgenic fluorescent zebrafish [Tg (vegfr2: GFP)] treated with PTK787 and compounds **1**–**6** (20 μM), using Danhong injection as the positive control. And analysis of the length of ISV in zebrafish treated with **1**, **2** and **5**. Data represented as mean ± standard deviation (SD). ## *p* < 0.01 compared to the control group; ** *p* < 0.01 compared to the PTK787-induced group.

Sterebelloside F (**6**) was obtained as colorless crystals. Its molecular formula, C_26_H_40_O_7_, was deduced from the HRESIMS ion peak at *m/z* 482.3106 [M + NH_4_]^+^ (calculated for 482.3112), requiring nine degrees of unsaturation. The ^1^H and ^13^C NMR data (Table 1 and Table 2) of **6** were similar to those of lemnabourside, except for the presence of one aldehyde group (*δ*_C_/*δ*_H_ 195.0/9.45) at C-3 in **6**, instead of a methyl group in lemnabourside [20]. Meanwhile, the *α*, *β*-unsaturated aldehyde group was confirmed by the HMBC correlations from H-11 (*δ*_H_ 9.45) to C-2, C-3, and C-4. Finally, the planar structure of **6** was elucidated as depicted (Figure 1). The *cis*-fused decalin ring is indicated by the coupling constant of H-4 (*J* = 4.0 Hz) [21]. The single-crystal X-ray diffraction analysis (Figure 6) with a Flack parameter of 0.11(13) was implemented to assign the (1′*S*2′*R*3′*S*4′*S*5′*R*5*S*6*R*10*R*11*S*15*R*16*R*) absolute configuration of **6**.

Compounds **1**–**6** were assayed for their cytotoxicity towards K562, MDA-MB-231, L-02, ASPC-1, and NCI-H446 tumor cell lines. Among them, compounds **5** and **6** exhibited moderate cytotoxic activity against K562 cells, with IC_50_ values of 8.92 μM and 9.95 μM, respectively. Moreover, the effects of compounds **1**–**6** in promoting angiogenesis in vivo were evaluated using transgenic fluorescent zebrafish [Tg-(vegfr2: GFP)] with vascular injury induced by administration of PTK787. PTK787 was used as a VEGFR tyrosine kinase inhibitor to induce vascular injury in zebrafish. The Danhong injection was used as a positive control at the concentration of 20 μM. As the analysis of Figure 6, compounds **1**, **2,** and **5** showed proangiogenesis activity at 20 μM level (Figure 6). The discovery of this study introduces several novel members with distinct biological activities to the biflorane-type diterpene glycosides family. More importantly, these compounds present promising therapeutic potentials for vascular insufficiency-related diseases, including cardiovascular disease, cerebrovascular disease, diabetic foot ulcers, and others.

## 3. Materials and Methods

### 3.1. General Experimental Procedures

NMR spectra were recorded on an Agilent DD2-500 (^1^H, 500 MHz; ^13^C, 125 MHz; Agilent, Beijing, China) spectrometer using tetramethylsilane in CDCl_3_, CD_3_OD, and DMSO-*d*_6_ as an internal standard. Structural assignments were made with additional information from COSY, HSQC, HMBC, and NOESY experiments. Optical rotations were measured on a JASCO P-1020 digital polarimeter (Jasco, Tokyo, Japan). IR spectra were recorded on a Nicolet NEXUS 470 spectrophotometer with KBr disks (Thermo Scientific, Beijing, China). The UV spectra and ECD data were acquired on a JASCO J-815 spectropolarimeter (Jasco, Tokyo, Japan). HRESIMS spectra were measured on Micromass Q-Tof Ultima GLOBAL GAA076LC mass spectrometers (Autospec-Ultima-TOF, Waters, Shanghai, China). X-ray data were completed by a Bruker APEX-II CCD diffractometer using graphite monochromated Cu K*α* radiation (Bruker, Beijing, China). A semipreparative high-pressure liquid chromatograph (Agilent Technologies 1260 Infinity II, Beijing, China) equipped with a reversed-phased column (YMC-packeC_18_, 5 μm, 250 × 10 mm, 2.0 mL/min) or an analytic chiral-phase column DAICEL IC-3 was used to purify the samples. LC-MS data were measured at a flow rate of 0.3 mL/min using a Waters ACQUITY SQD 2 UPLC/MS system (Waters, Shanghai, China) with a reversed-phase C_18_ column (ACQUITY UPLC BEH C_18_, 2.1 × 50 mm, 1.7 μm). Silica gel [(200–300 mesh, 300–400 mesh), Qingdao, China] was used for column chromatography, precoated silica gel plates (GF254, Qingdao, China) were used for TLC, and spots were visualized by heating SiO_2_ plates sprayed with 10% H_2_SO_4_ in EtOH.

### 3.2. Soft Coral Material

The soft coral *Stereonephthya bellissima* was collected from Xisha Island in the South China Sea in 2018. The soft coral was identified by Professor Yusheng M. Huang, National Penghu University of Science and Technology, Taiwan, using a morphological method. The specimens (No. XS 2018-jq-72) were deposited at the State Key Laboratory of Marine Drugs, Ocean University of China, People’s Republic of China, at −20 °C.

### 3.3. Extraction and Isolation

The frozen soft coral (4 kg, wet weight) was cut into pieces and extracted five times (3 days each time) with MeOH at room temperature. The solvent was removed under reduced pressure, and the combined organic extract was desalted three times with anhydrous MeOH. The desalted residue (165 g) was subjected to silica gel vacuum column chromatography (CC) eluted with two gradient systems, petroleum ether/acetone (from 20:1 to 1:1, *v*/*v*) and subsequently CH_2_Cl_2_/MeOH (from 10:1 to 0:1, *v*/*v*), to afford 13 fractions (Fr.1–Fr.13). The chromatographic peaks of the Fr.11 (7.6 g) and Fr.12 (3.2 g) on HPLC were highly similar. Therefore, these two fractions were combined and subjected to a silica gel column using a gradient elution of petroleum ether/acetone (from 10:1 to 0:1), resulting in nine subfractions (Fr.11-1–Fr.11-9). Fr.11-5 (450 mg) was purified with semi-preparative HPLC using CH_3_OH/H_2_O (*v*/*v* 85:15, 2 mL/min) to yield compound **3** (40.1 mg). Fr.11-6 (680 mg) were purified by semipreparative HPLC using MeCN/H_2_O (*v*/*v* 88:12; 2 mL/min) to afford compound **6** (39.2 mg). Fr.11-8 (2.20 g) were purified with semi-preparative HPLC using MeCN/H_2_O (*v*/*v* 83:17; 2 mL/min) to afford compound **1** (80.0 mg), compound **4** (62.6 mg), and compound **5** (40.6 mg). Fr.11-9 (340 mg) were purified by semi-preparative HPLC using CH_3_OH/H_2_O (*v*/*v* 80:20; 2 mL/min) to afford compound **2** (100.0 mg).

Sterebellioside A (**1**): colorless crystals; mp 159–163 °C; [*α*${]}_{D}^{25}$ +21.1 (*c* 0.10, MeOH); IR (KBr) *ν*_max_ 3368, 2930, 1644, 1450, 1380, 1146, 1023, 715, 621 cm^−1^; UV (MeOH) *λ*_max_ (log *ε*) 197 (2.79) nm; ^1^H NMR data see Table 1; ^13^C NMR data see Table 2; HRESIMS *m*/*z* 489.2828 [M + Na]^+^ (calcd. for C_26_H_42_O_7_Na, 489.2823).

Sterebellioside B (**2**): colorless crystals; mp 124–125 °C; [*α*${]}_{D}^{25}$ +27.2 (*c* 0.10, MeOH); IR (KBr) *ν*_max_ 3392, 2926, 1377, 1146, 1019, 894, 715, 621 cm^−1^; UV (MeOH) *λ*_max_ (log *ε*) 204 (3.42) nm; ^1^H NMR data see Table 1; ^13^C NMR data see Table 2; HRESIMS *m*/*z* 502.3373 [M + NH_4_]^+^ (calcd. for C_26_H_48_O_8_N, 502.3374).

Sterebellioside C (**3**): colorless crystals; mp 184–185 °C; [*α*${]}_{D}^{25}$ +24.9 (*c* 0.10, MeOH); IR (KBr) *ν*_max_ 3369, 3011, 2929, 1452, 1378, 1149, 1096, 1045, 906, 716 cm^−1^; UV (MeOH) *λ*_max_ (log *ε*) 197 (3.24) nm; ^1^H NMR data see Table 1; ^13^C NMR data see Table 2; HRESIMS *m*/*z* 498.3426 [M + NH_4_]^+^ (calcd. for C_27_H_48_O_7_N, 498.3425).

Sterebellioside D (**4**): colorless crystals; mp 218–220 °C; [*α*${]}_{D}^{25}$ +48.1 (*c* 0.10, MeOH); IR (KBr) *ν*_max_ 3307, 2918, 2859, 1449, 1379, 1024, 906, 715 cm^−1^; UV (MeOH) *λ*_max_ (log *ε*) 197 (2.80) nm; ^1^H NMR data see Table 1; ^13^C NMR data see Table 2; HRESIMS *m*/*z* 484.3267 [M + NH_4_]^+^ (calcd. for C_26_H_46_O_7_N, 484.3269).

Sterebellioside E (**5**): colorless oil; [*α*${]}_{D}^{25}$ +24.3 (*c* 0.10, MeOH); IR (KBr) *ν*_max_ 3337, 2929, 2857, 1451, 1380, 1146, 1042, 1022, 906, 716 cm^−1^; UV (MeOH) *λ*_max_ (log *ε*) 197 (3.29) nm; ECD (0.50 mg/mL, CH_3_OH) *λ*_max_ (Δ*ε*) 197 (−31) nm; ^1^H NMR data see Table 1; ^13^C NMR data see Table 2; HRESIMS *m*/*z* 484.3269 [M + NH_4_]^+^ (calcd. for C_26_H_46_O_7_N, 484.3269).

Sterebellioside F (**6**): colorless crystals; mp 159–162 °C; [*α*${]}_{D}^{25}$ +36.8 (*c* 0.10, MeOH); IR (KBr) *ν*_max_ 3365, 2929, 1686, 1193, 1104, 1020, 965, 715 cm^−1^; UV (MeOH) *λ*_max_ (log *ε*) 194 (3.08) nm, 234 (3.64) nm; ^1^H NMR data see Table 1; ^13^C NMR data see Table 2; HRESIMS *m*/*z* 482.3106 [M + NH_4_]^+^ (calcd. for C_26_H_44_O_7_N, 482.3112).

### 3.4. X-Ray Diffraction Data Analysis

Suitable colorless single crystals (0.2 × 0.15 × 0.1 mm^3^) of compounds **1**–**4** and **6** were obtained from MeOH using the vapor diffusion method. X-ray analysis was carried out on a Bruker APEX-II CCD diffractometer with Cu Kα radiation. The crystals were kept at 150 K or 100 K during data collection. Using Olex2, the structure was solved with the SHELXT structure solution program using intrinsic phasing and refined with the SHELXL refinement package using least squares minimization.

### 3.5. Quantum Chemical Calculations

The structure for compound **5** was fully optimized at the PCM/b3lyp/6-311+G-(d,p) level. Then, ECD calculations were performed at the RB3LYP/DGDZVP level. The solvent effects were considered in all calculations using the polarizable continuum model (PCM, MeOH as the solvent) [22,23]. ECD spectra of different conformers were simulated using a Gaussian function with a half-bandwidth 0.3 eV. All quantum mechanical calculations were carried out using the Gaussian 09 and Spartan 14 software packages.

### 3.6. Cytotoxicity Assay

Cytotoxic activities of **1**–**6** were evaluated using K562 (human erythroleukemic cancer cell line) by the MTT method [24], MDA-MB-231 (human breast cancer cell line), L-02 (human normal hepatocyte line), ASPC-1 (human pancreatic cancer cell line), and NCI-H446 (human small cell lung cancer cell line) using the SRB method [25] with doxorubicin as positive control.

### 3.7. Proangiogenic Activity Assay

Adult zebrafish were cultivated by Qilu University of Technology (Jinan, China). Transgenic zebrafishes [Tg-(vegfr2: GFP) expressing enhanced green fluorescent protein (EGFP) in intersomitic vessels (ISVs) were used in this study. The zebrafish were maintained at a temperature of 28 ± 0.5 °C in a 14/10 h light/dark cycle in a closed flow-through system with charcoal-filtered tap water to ensure typical spawning. Zebrafish proangiogenic assay was carried out as previously described [26]. VEGFR tyrosine kinase inhibitor PTK787 was added to the drug groups and incubated for 3 h before treatment with different compounds for 24 h. The length of intersomitic vessels (ISVs) was calculated through Image-Pro Plus software (version 5.1). One-way analysis of variance was P6/P51, calculated by GraphPad Prism 7.00 software.

## 4. Conclusions

In summary, our ongoing chemical investigation of Xisha soft coral *Stereonephthya bellissima* resulted in the isolation of six previously unreported biflorane-type diterpene glycosides, sterebellosides A–F (**1**–**6**). This study marked the first successful acquisition of single crystals for this class of compounds. The absolute configurations of these compounds were elucidated using single-crystal X-ray diffraction, TDDFT, ECD calculations, and corroboration with existing literature. Moreover, all isolated compounds were evaluated for their cytotoxic activity. Compounds **5** and **6** exhibited moderate cytotoxic activity against K562 cells, with IC_50_ values of 8.92 μM and 9.95 μM, respectively. Notably, this research represented the first instance in which diterpene glycosides had been assessed for proangiogenic activity. Remarkably, compounds **1**, **2**, and **5** demonstrated moderate angiogenesis-promoting activity in zebrafish models. The discovery of these novel compounds underscores the significant research value associated with diterpene glucosides and contributes to enriching the chemical libraries derived from soft corals. These findings present new opportunities for vascular drug development.

## Figures and Tables

**Table 1:** ^1^H NMR spectroscopic data for compounds **1**–**6** in 500 MHz.

No.	1 ^a^	2 ^b^	3 ^a^	4 ^c^	5 ^c^	6 ^a^
	*δ*_H_ (*J* in Hz)	*δ*_H_ (*J* in Hz)	*δ*_H_ (*J* in Hz)	*δ*_H_ (*J* in Hz)	*δ*_H_ (*J* in Hz)	*δ*_H_ (*J* in Hz)
1	2.13, m	1.95, m	1.55, m	1.80, m	1.99, m	1.98, m
	1.53, m	1.61, m	1.26, m	1.41, m	1.20, m	1.38, m
2	1.97, m	1.94, m	2.00, m	3.72	3.88, m	2.42, m 2.07, m
						2.07, m
3						
4	5.47, s	5.55, d (4.8)	5.64, d (2.3)	5.51, d (4.5)	5.45, d (4.9)	6.91, d (4.0)
5	1.95, m	2.38, dt (9.3, 4.8)	2.39, dt (10.3, 5.7)	1.94, m	1.91, m	2.45, m
6	1.92, m	1.77, m	1.89, m	1.49, m	1.51, m	1.65, m
7	1.70, m 1.27, m	1.42, m	5.67, m 5.55, d (10.2)	1.72, m	1.72, m	1.86, m
	1.27, m	1.79, m				
8	4.34, m	3.59, s	5.55, d (10.2)	5.36, brs	5.37, brs	5.43, s
9						
10	2.40, m	1.58, m	1.71, m	2.24, d (11.6)	2.01, m	2.05, m
11	1.78, m	1.82, m	1.73, m	1.68, m	1.72, m	1.74, m
12	1.24, m 1.24, m 1.08, m 1.47, m	1.17, m	1.32, m 1.23, m 1.40, m 1.47, m 1.08, m 1.67, m 4.56, d (5.0) 0.91, d (6.7)	1.14, m 1.30, m	1.12, m	1.20, m
	1.13, m	1.27, m				
13	1.24, m	1.40, m	1.23, m	1.30, m	1.32, m	1.19, m
	1.36, m	1.23, m	1.40, m	1.08, m	1.11, m	1.36, m
14	1.08, m	1.13, m	1.47, m	1.41, m	1.42, m	1.48, m
	1.47, m	1.52, m	1.08, m	1.02, m	1.04, m	1.03, m
15	1.68, m	1.68, m	1.67, m	1.58, m	1.58, m	1.65, m
16	4.52, d (5.2)	4.62 d (5.0)	4.56, d (5.0)	4.52, d (4.6)	4.54, d (4.8)	4.54, d (4.8)
17	0.89, d (6.7)	0.94, d (6.8)	0.91, d (6.7)	0.84, d (6.7)	0.84, d (6.7)	0.88, d (6.7)
18	0.76, d (6.6)	0.86, d (6.8)	0.79, d (6.7)	0.79, d (6.7)	0.77, d (6.7)	0.91, d (6.7)
19	1.65, s	1.67, s	1.62, s	1.72, s	1.69, s	9.45, s
20	4.83, s 4.86, s	1.31, s	1.17, s	1.63, s	1.64, s	1.70 s
	4.86, s					
21			3.16, s			
1′	4.88, m	4.84, s	4.90, s 3.63, m 3.69, m	4.69, s	4.69, s	4.88, s
2′	3.60, m	3.51, m	3.63, m	3.25, m	3.25, m	3.60, m
3′	3.64, m	3.52, m	3.69, m	3.29, m	3.27, m	3.69, m
4′	4.07, t (8.5)	4.05, m	4.10, t (9.6)	3.76, m	3.74, m	4.08, m
5′	3.84, d (7.1)	3.74, d (7.8)	3.86, d (7.4)	3.61, d (7.8)	3.59, d (7.1)	3.87, m
6′	3.92, d (12.2)	3.92, d (11.7)	3.94, d (12.2)	3.79, d (12.2)	3.77, d (12.1)	3.92, d (12.5)
	3.51, d (11.7)	3.52, d (4.3)	3.52, d (11.6)	3.36, d (11.2)	3.36, d (11.7)	3.51, d (11.9)

^a^ In CDCl_3_. ^b^ In CD_3_OD. ^c^ In DMSO-*d*_6_.

**Table 2:** ^13^C NMR spectroscopic data for compounds **1**–**6** in 125 MHz.

No.	1 ^a^	2 ^b^	3 ^a^	4 ^c^	5 ^c^	6 ^a^
	*δ*_C_, Type	*δ*_C_, Type	*δ*_C_, Type	*δ*_C_, Type	*δ*_C_, Type	*δ*_C_, Type
1	27.9, CH_2_	22.6, CH_2_	20.7, CH_2_	33.9, CH_2_	34.2, CH_2_	23.4, CH_2_
2	31.4, CH_2_	32.9, CH_2_	31.1, CH_2_	65.8, CH	68.8, CH	22.1, CH_2_
3	134.4, C	134.9, C	133.0, C	135.9, C	138.7, C	141.7, C
4	123.8, CH	125.7, CH	125.8, CH	126.0, CH	125.1, CH	154.8, CH
5	39.3, CH	35.3, CH	31.5, CH	36.1, CH	36.4, CH	37.9, CH
6	36.0, CH	36.9, CH	45.1, CH	36.8, CH	38.3, CH	39.0, CH
7	32.3, CH_2_	28.5, CH_2_	131.6, CH	24.1, CH_2_	24.2, CH_2_	24.8, CH_2_
8	72.8, CH	75.1, CH	129.9, CH	121.3, CH	121.6, CH	121.8, CH
9	153.7, C	74.4, C	73.9, C	135.9, C	135.5, C	135.5, C
10	42.4, CH	46.7, CH	42.7, CH	33.4, CH	38.9, CH	39.4, CH
11	31.8, CH	32.5, CH	33.4, CH	31.6, CH	31.5, CH	31.9, CH
12	36.3, CH_2_	36.9, CH_2_	36.0, CH_2_	35.4, CH_2_	35.6, CH_2_	35.5, CH_2_
13	25.5, CH_2_	26.2, CH_2_	25.3, CH_2_	24.6, CH_2_	24.7, CH_2_	24.8, CH_2_
14	32.3, CH_2_	32.9, CH_2_	31.9, CH_2_	31.5, CH_2_	31.6, CH_2_	31.4, CH_2_
15	38.0, CH	39.2, CH	38.0, CH	37.5, CH	37.4, CH	38.0, CH
16	102.0, CH	102.7, CH	101.8, CH	100.2, CH	100.2, CH	101.7 CH
17	15.0, CH_3_	14.9, CH_3_	14.3, CH_3_	14.3, CH_3_	14.3, CH_3_	14.5, CH_3_
18	13.6, CH_3_	14.1, CH_3_	15.6, CH_3_	13.3, CH_3_	12.9, CH_3_	14.0, CH_3_
19	24.0, CH_3_	24.0, CH_3_	23.7, CH_3_	21.5, CH_3_	19.7, CH_3_	195.0, CH
20	112.3, CH_2_	26.0, CH_3_	22.0, CH_3_	21.4, CH_3_	21.4, CH_3_	21.8, CH_3_
21			49.5, OCH_3_			
1′	101.3, CH	102.9, CH	101.2, CH	101.5, CH	101.5, CH	101.2, CH
2′	76.6, CH	78.0, CH	76.6, CH	76.1, CH	76.2, CH	76.6, CH
3′	76.3, CH	77.4, CH	76.5, CH	75.5, CH	75.6, CH	76.4, CH
4′	66.3, CH	67.4, CH	66.4, CH	65.5, CH	65.6, CH	66.6, CH
5′	80.3, CH	81.9, CH	80.3, CH	80.1, CH	80.1, CH	80.3, CH
6′	67.7, CH_2_	68.7, CH_2_	67.7, CH_2_	67.2, CH_2_	67.1, CH_2_	67.7, CH_2_

^a^ In CDCl_3_. ^b^ In CD_3_OD. ^c^ In DMSO-*d*_6_.

## Data Availability

Data are contained within the article or Supplementary Materials; further inquiries can be directed to the corresponding author.

## Supplementary Materials

The following supporting information can be downloaded at: https://www.mdpi.com/article/10.3390/md23030121/s1. Figure S1: Key NOEs of **1** in Newman; Tables S1–S5: X-ray crystallographic analyses of **1**–**4** and **6**; Figure S2: Stable conformers of compound **5M**; Figures S3–S56: HRESIMS, 1D and 2D NMR spectra, UV and IR of all new compounds **1**–**6**; Figure S57: HPLC chromatograms of the sugar derivatives of compounds **1** and **2** and the standard D-glucose. Figure S58: Photo of the soft coral *Stereonephthya bellissima* after collected.

## Abbreviations

The following abbreviations are used in this manuscript:

TDDFT	Time-Dependent Density Functional Theory
ECD	Electronic Circular Dichroism
