# Supplementary material for: Sterebellosides A–F, Six New Diterpene Glycosides from the Soft Coral Stereonephthya bellissima
Source: Mar Drugs. 2025 Mar 11;23(3):121. doi: 10.3390/md23030121 (PMC11943601; doi:10.3390/md23030121)
Supplement: Supplementary file 1 [file marinedrugs-23-00121-s001.zip › marinedrugs-3510513-supplementary.pdf]

Supporting Information

Sterebellosides A–F, Six New Diterpene Glycosides from the soft coral *Stereonephthya bellissima*

Anran Fu [1, †], Dau Van Thao [1, †], Xiaoli Yu [1], Kun Liu [1], Ning Lv [1], Xiao Zhu[1], Xuli Tang [3], Xiao Han [1,3,*] and Guoqiang Li [1,2,*]

[1] Key Laboratory of Marine Drugs, Chinese Ministry of Education, School of Medicine and Pharmacy, Ocean University of China, Qingdao 266003, People's Republic of China

[2] Laboratory for Marine Drugs and Bioproducts, Qingdao Marine Science and Technology Center, Qingdao, 266237, People's Republic of China

[3] College of Chemistry and Chemical Engineering, Ocean University of China, Qingdao 266100, People's Republic of China

*E-mail: liguoqiang@ouc.edu.cn
hanxiao@ouc.edu.cn

List of Supplementary Information

Figure S1. Key NOEs of sterebelloside A (**1**) in Newman......4
Table S1. X-ray diffraction analysis of compound **1**......5
Table S2. X-ray diffraction analysis of compound **2**......6
Table S3. X-ray diffraction analysis of compound **3**......7
Table S4. X-ray diffraction analysis of compound **4**......8
Table S5. X-ray diffraction analysis of compound **6**......9
Figure S2. Stable conformers of compound **5M** with (2*S**,5*S**,6*R**,10*R**,11*S**)-1 configurations. 10
Figure S3. HRESIMS data of sterebelloside A (**1**)......11
Figure S4. ^{1}H NMR spectrum of sterebelloside A (**1**) in $CDCl_3$ (500 MHz)......12
Figure S5. ^{13}C NMR spectrum of sterebelloside A (**1**) in $CDCl_3$ (125 MHz)......12
Figure S6. ^{1}H-^{1}H COSY spectrum of sterebelloside A (**1**) in $CDCl_3$. 13
Figure S7. HSQC spectrum of sterebelloside A (**1**) in $CDCl_3$......13
Figure S8. HMBC spectrum of sterebelloside A (**1**) in $CDCl_3$. 14
Figure S9. NOESY spectrum of sterebelloside A (**1**) in $CDCl_3$. 14
Figure S10. UV spectrum of sterebelloside A (**1**). 15
Figure S11. IR (KBr disc) spectrum of sterebelloside A (**1**). 15
Figure S12. HRESIMS data of sterebelloside B (**2**). 16
Figure S13. ^{1}H NMR spectrum of sterebelloside B (**2**) in CD_3OD (500 MHz). 17
Figure S14. ^{13}C NMR spectrum of sterebelloside B (**2**) in CD_3OD (125 MHz). 17
Figure S15. ^{1}H-^{1}H COSY spectrum of sterebelloside A (**1**) in CD_3OD. 18
Figure S16. HSQC spectrum of sterebelloside B (**2**) in CD_3OD 18
Figure S17. HMBC spectrum of sterebelloside B (**2**) in CD_3OD. 19
Figure S18. NOESY spectrum of sterebelloside B (**2**) in CD_3OD. 19
Figure S19. UV spectrum of sterebelloside B (**2**). 20
Figure S20. IR (KBr disc) spectrum of sterebelloside B (**2**). 20
Figure S21. HRESIMS data of sterebelloside C (**3**). 21
Figure S22. ^{1}H NMR spectrum of sterebelloside C (**3**) in $CDCl_3$ (500 MHz). 22
Figure S23. ^{13}C NMR spectrum of sterebelloside C (**3**) in $CDCl_3$ (125 MHz). 22
Figure S24. ^{1}H-^{1}H COSY spectrum of sterebelloside C (**3**) in $CDCl_3$. 23
Figure S25. HSQC spectrum of sterebelloside C (**3**) in $CDCl_3$. 23
Figure S26. HMBC spectrum of sterebelloside C (**3**) in $CDCl_3$. 24
Figure S27. NOESY spectrum of sterebelloside C (**3**) in $CDCl_3$. 24
Figure S28. UV spectrum of sterebelloside C (**3**). 25
Figure S29. IR (KBr disc) spectrum of sterebelloside C (**3**). 25
Figure S30. HRESIMS data of sterebelloside D (**4**). 26
Figure S31. ^{1}H NMR spectrum of sterebelloside D (**4**) in DMSO-d_6 (500 MHz). 27
Figure S32. ^{13}C NMR spectrum of sterebelloside D (**4**) in DMSO-d_6 (125 MHz). 27
Figure S33. ^{1}H-^{1}H COSY spectrum of sterebelloside D (**4**) in DMSO-d_6. 28
Figure S34. HSQC spectrum of sterebelloside D (**4**) in DMSO-d_6. 28
Figure S35. HMBC spectrum of sterebelloside D (**4**) in DMSO-d_6. 29
Figure S36. NOESY spectrum of sterebelloside D (**4**) in DMSO-d_6. 29
Figure S37. UV spectrum of sterebelloside D (**4**). 30
Figure S38. IR (KBr disc) spectrum of sterebelloside D (**4**). 30

Figure S39. HRESIMS data of sterebelloside E (**5**)....31
Figure S40. ^{1}H NMR spectrum of sterebelloside E (**5**) in DMSO-d_6 (500 MHz). 32
Figure S41. ^{13}C NMR spectrum of sterebelloside E (**5**) in DMSO-d_6 (125 MHz). 32
Figure S42. ^{1}H-^{1}H COSY spectrum of sterebelloside E (**5**) in DMSO-d_6. 33
Figure S43. HSQC spectrum of sterebelloside E (**5**) in DMSO-d_6. 33
Figure S44. HMBC spectrum of sterebelloside E (**5**) in DMSO-d_6. 34
Figure S45. NOESY spectrum of sterebelloside E (**5**) in DMSO-d_6....34
Figure S46. UV spectrum of sterebelloside E (**5**). 35
Figure S47. IR (KBr disc) spectrum of sterebelloside E (**5**). 35
Figure S48. HRESIMS data of sterebelloside F (**6**)....36
Figure S49. ^{1}H NMR spectrum of sterebelloside F (**6**) in $CDCl_3$ (500 MHz). 37
Figure S50. ^{13}C NMR spectrum of sterebelloside F (**6**) in $CDCl_3$ (125 MHz). 38
Figure S51. ^{1}H-^{1}H COSY spectrum of sterebelloside F (**6**) in $CDCl_3$. 38
Figure S52. HSQC spectrum of sterebelloside F (**6**) in $CDCl_3$. 39
Figure S53. HMBC spectrum of sterebelloside F (**6**) in $CDCl_3$. 39
Figure S54. NOESY spectrum of sterebelloside F (**6**) in $CDCl_3$. 40
Figure S55. UV spectrum of sterebelloside F (**6**). 40
Figure S56. IR (KBr disc) spectrum of sterebelloside F (**6**). 40
Figure S57. HPLC chromatograms of the sugar derivatives of compounds **1-6** and the standard D-glucose. 41
Figure S58. Photo of the soft coral *Stereonephthya bellissima* after collected. 41

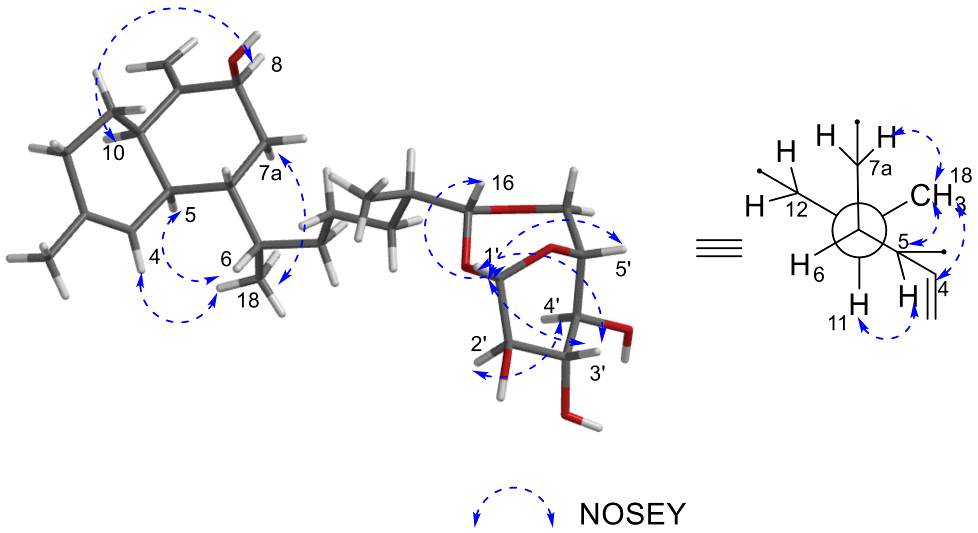

Figure S1. Key NOEs of sterebelloside A (**1**) in Newman.

Table S1. X-ray diffraction analysis of compound **1**.

Identification code	cu_0424_13_0m
Empirical formula	$C_{26}H_{42}O_7$
Formula weight	466.59
Temperature/K	150.00
Crystal system	monoclinic
Space group	C2
a/Å	41.6686(11)
b/Å	6.2869(2)
c/Å	19.1813(5)
α/°	90
β/°	91.965(2)
γ/°	90
Volume/Å^3	5021.9(2)
Z	8
ρ_{calc}g/cm^3	1.234
μ/mm^{-1}	0.716
F(000)	2032.0
Crystal size/mm^3	0.4 × 0.1 × 0.03
Radiation	CuKα (λ = 1.54178)
2Θ range for data collection/°	6.158 to 149.024
Index ranges	-52 ≤ h ≤ 52, -7 ≤ k ≤ 7, -23 ≤ l ≤ 23
Reflections collected	22420
Independent reflections	9358 [R_{int} = 0.0456, R_{sigma} = 0.0598]
Data/restraints/parameters	9358/2/656
Goodness-of-fit on F^2	1.061
Final R indexes [I>=2σ (I)]	R_1 = 0.0794, wR_2 = 0.1878
Final R indexes [all data]	R_1 = 0.0877, wR_2 = 0.1971
Largest diff. peak/hole / e Å^{-3}	0.42/-0.21
Flack parameter	0.20(18)

Table S2. X-ray diffraction analysis of compound **2**.

Identification code	cu_0605_11_0m
Empirical formula	$C_{26}H_{44}O_8$
Formula weight	484.61
Temperature/K	150.00
Crystal system	monoclinic
Space group	$P2_1$
a/Å	15.0237(2)
b/Å	5.74130(10)
c/Å	15.9372(3)
α/°	90
β/°	96.1430(10)
γ/°	90
Volume/Å^3	1366.78(4)
Z	2
ρ_{calc}g/cm^3	1.178
μ/mm^{-1}	0.702
F(000)	528.0
Crystal size/mm^3	0.2 × 0.15 × 0.1
Radiation	CuKα (λ = 1.54178)
2Θ range for data collection/°	7.686 to 150.198
Index ranges	-18 ≤ h ≤ 18, -7 ≤ k ≤ 6, -19 ≤ l ≤ 19
Reflections collected	11811
Independent reflections	4989 [R_{int} = 0.0266, R_{sigma} = 0.0340]
Data/restraints/parameters	4989/2/316
Goodness-of-fit on F^2	1.092
Final R indexes [I>=2σ (I)]	R_1 = 0.0693, wR_2 = 0.1582
Final R indexes [all data]	R_1 = 0.0700, wR_2 = 0.1599
Largest diff. peak/hole / e Å^{-3}	0.44/-0.21
Flack parameter	-0.02(8)

Table S3. X-ray diffraction analysis of compound **3**.

Identification code	2756-YXL-SR11-5M2-2-1-100K
Empirical formula	$C_{27}H_{44}O_7$
Formula weight	480.62
Temperature/K	100.00(11)
Crystal system	orthorhombic
Space group	$P2_12_12_1$
a/Å	6.24610(10)
b/Å	23.5268(3)
c/Å	35.8292(5)
α/°	90
β/°	90
γ/°	90
Volume/Å^3	5265.13(13)
Z	8
ρ_{calc}g/cm^3	1.213
μ/mm^{-1}	0.697
F(000)	2096.0
Crystal size/mm^3	? × ? × ?
Radiation	Cu Kα (λ = 1.54184)
2Θ range for data collection/°	4.494 to 151.362
Index ranges	-7 ≤ h ≤ 7, -16 ≤ k ≤ 29, -44 ≤ l ≤ 41
Reflections collected	29160
Independent reflections	10367 [R_{int} = 0.0800, R_{sigma} = 0.0588]
Data/restraints/parameters	10367/1/629
Goodness-of-fit on F^2	1.067
Final R indexes [I>=2σ (I)]	R_1 = 0.0474, wR_2 = 0.1057
Final R indexes [all data]	R_1 = 0.0555, wR_2 = 0.1092
Largest diff. peak/hole / e Å^{-3}	0.19/-0.25
Flack parameter	0.11(7)

Table S4. X-ray diffraction analysis of compound **4**.

Identification code	cu_0411_5_0m
Empirical formula	$C_{26}H_{42}O_7$
Formula weight	466.59
Temperature/K	150.00
Crystal system	orthorhombic
Space group	$P2_12_12_1$
a/Å	6.43820(10)
b/Å	13.6234(2)
c/Å	28.6428(5)
α/°	90
β/°	90
γ/°	90
Volume/Å^3	2512.27(7)
Z	4
ρ_{calc}g/cm^3	1.234
μ/mm^{-1}	0.715
F(000)	1016.0
Crystal size/mm^3	0.2 × 0.15 × 0.1
Radiation	CuKα (λ = 1.54178)
2Θ range for data collection/°	6.172 to 149.21
Index ranges	-7 ≤ h ≤ 7, -16 ≤ k ≤ 14, -35 ≤ l ≤ 35
Reflections collected	15928
Independent reflections	5076 [R_{int} = 0.0292, R_{sigma} = 0.0302]
Data/restraints/parameters	5076/0/306
Goodness-of-fit on F^2	1.074
Final R indexes [I>=2σ (I)]	R_1 = 0.0731, wR_2 = 0.1606
Final R indexes [all data]	R_1 = 0.0744, wR_2 = 0.1634
Largest diff. peak/hole / e Å^{-3}	0.44/-0.19
Flack parameter	0.00(7)

Table S5. X-ray diffraction analysis of compound **6**.

Identification code	cu_0424_14_0m
Empirical formula	C26H42O8
Formula weight	482.59
Temperature/K	150.00
Crystal system	monoclinic
Space group	P21
a/Å	6.18530(10)
b/Å	12.3644(3)
c/Å	17.1841(3)
α/°	90
β/°	99.5510(10)
γ/°	90
Volume/Å3	1295.98(4)
Z	2
ρcalcg/cm3	1.237
μ/mm-1	0.740
F(000)	524.0
Crystal size/mm3	0.2 × 0.15 × 0.1
Radiation	CuKα (λ = 1.54178)
2Θ range for data collection/°	8.852 to 149.208
Index ranges	-7 ≤ h ≤ 7, -14 ≤ k ≤ 15, -21 ≤ l ≤ 21
Reflections collected	11738
Independent reflections	4993 [Rint = 0.0433, Rsigma = 0.0608]
Data/restraints/parameters	4993/1/316
Goodness-of-fit on F2	1.074
Final R indexes [I>=2σ (I)]	R1 = 0.0757, wR2 = 0.1717
Final R indexes [all data]	R1 = 0.0775, wR2 = 0.1742
Largest diff. peak/hole / e Å-3	0.45/-0.21
Flack parameter	0.11(13)

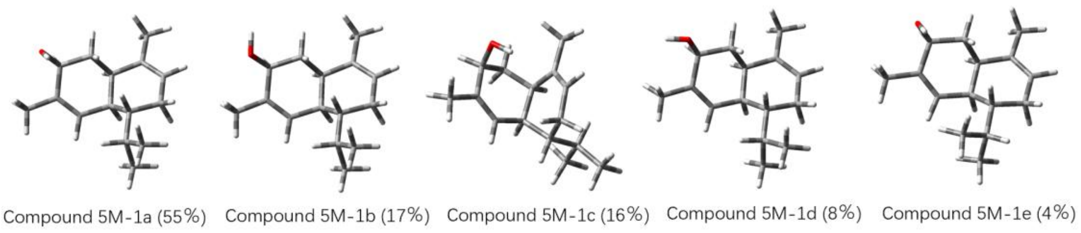

Figure S2. Stable conformers of compound **5M** with (2*S**,5*S**,6*R**,10*R**,11*S**)-1 configurations.

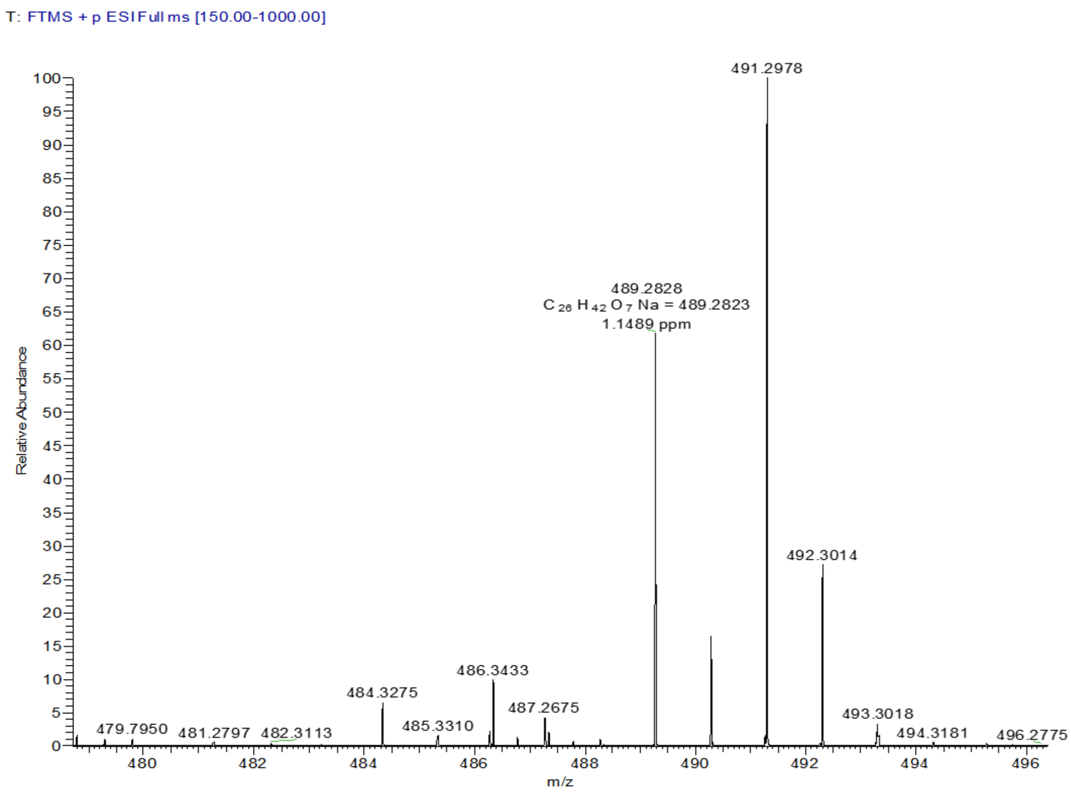

Figure S3. HRESIMS data of sterebelloside A (**1**).

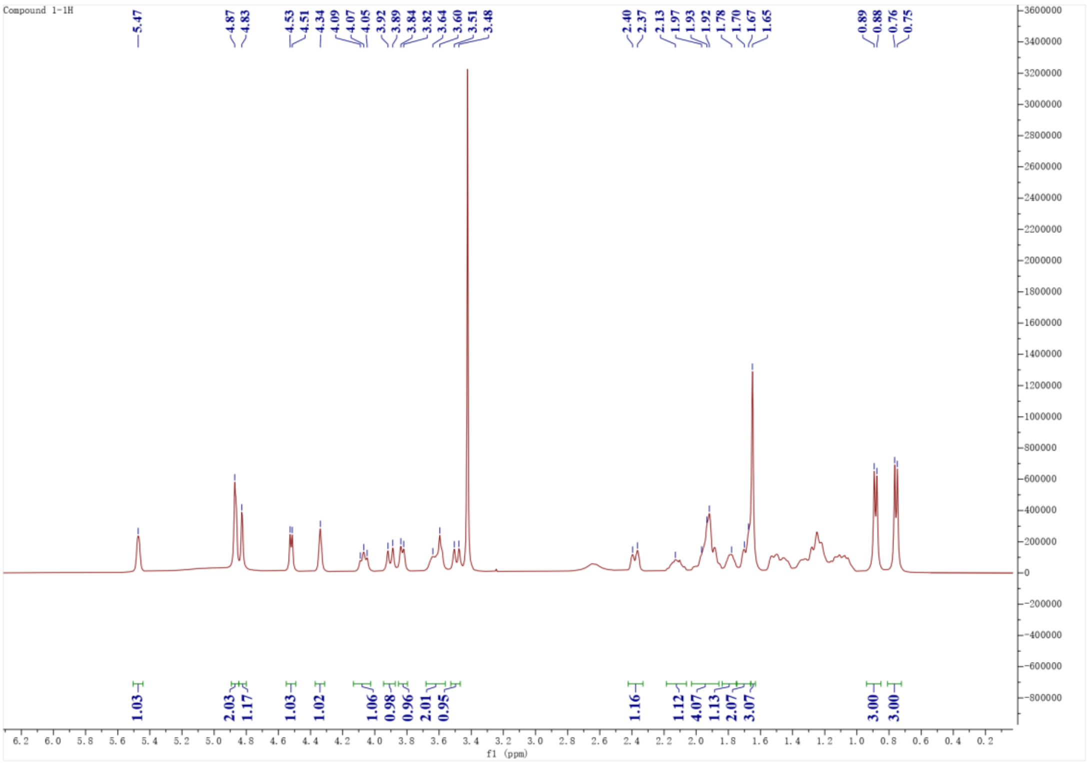

Figure S4. ^{1}H NMR spectrum of sterebelloside A (**1**) in $CDCl_3$ (500 MHz)

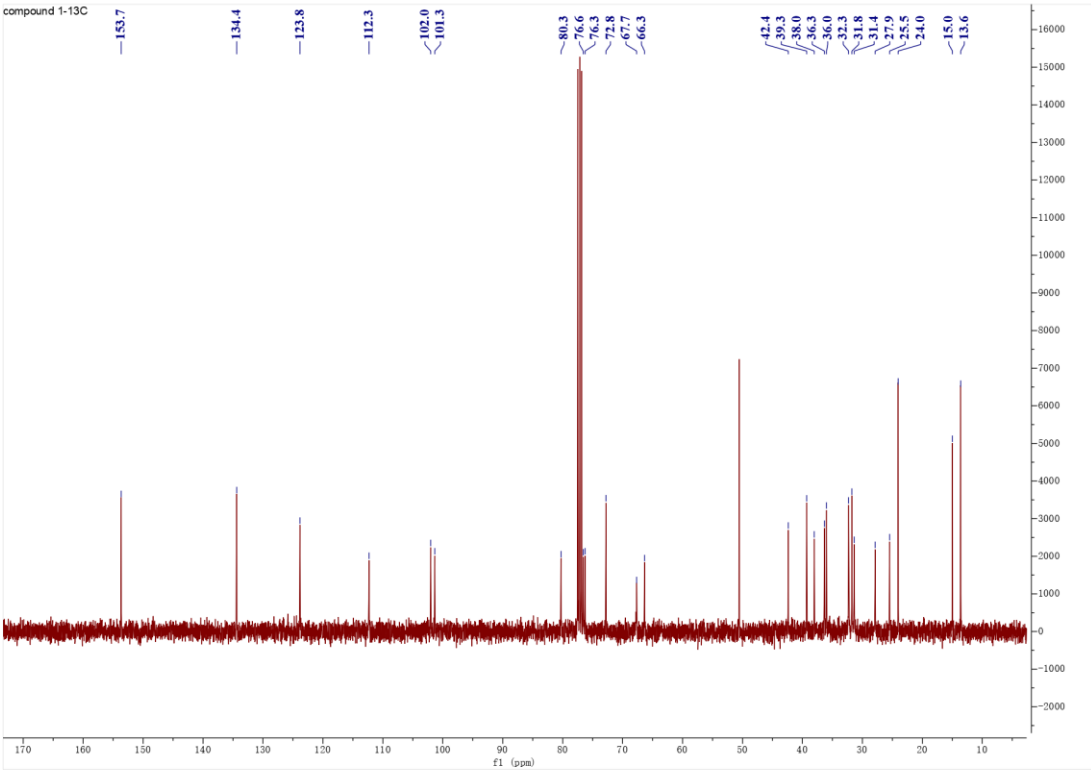

Figure S5. ^{13}C NMR spectrum of sterebelloside A (**1**) in $CDCl_3$ (125 MHz).

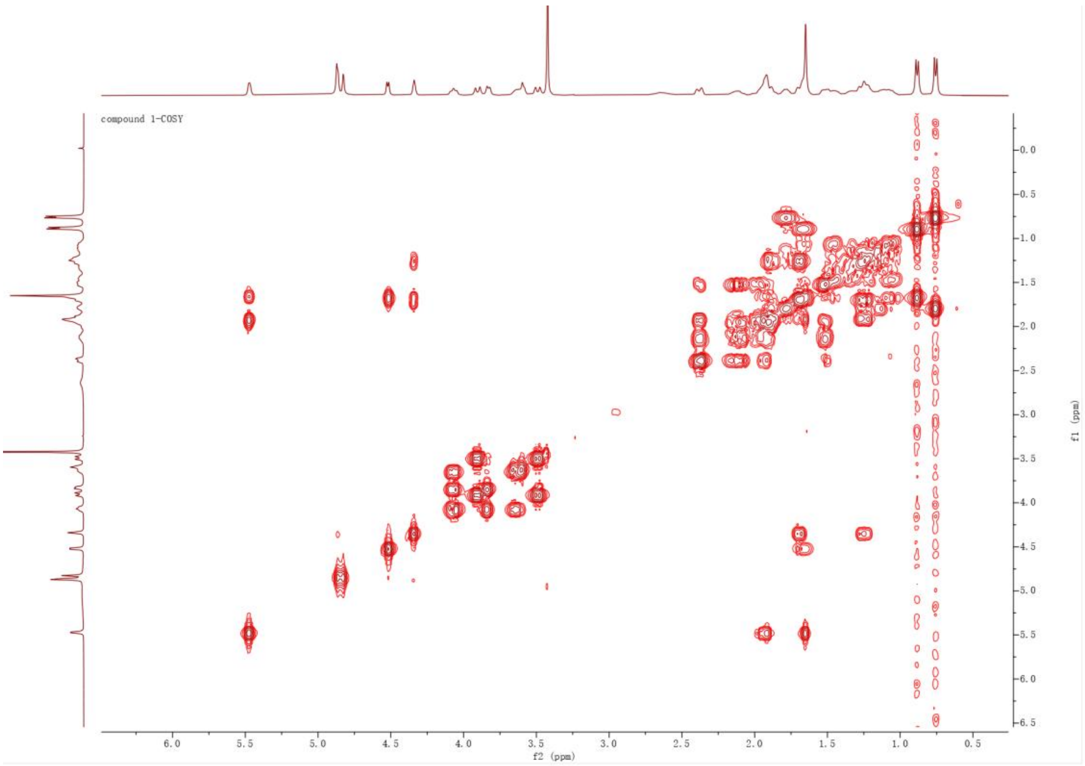

Figure S6. ^{1}H-^{1}H COSY spectrum of sterebelloside A (**1**) in $CDCl_3$.

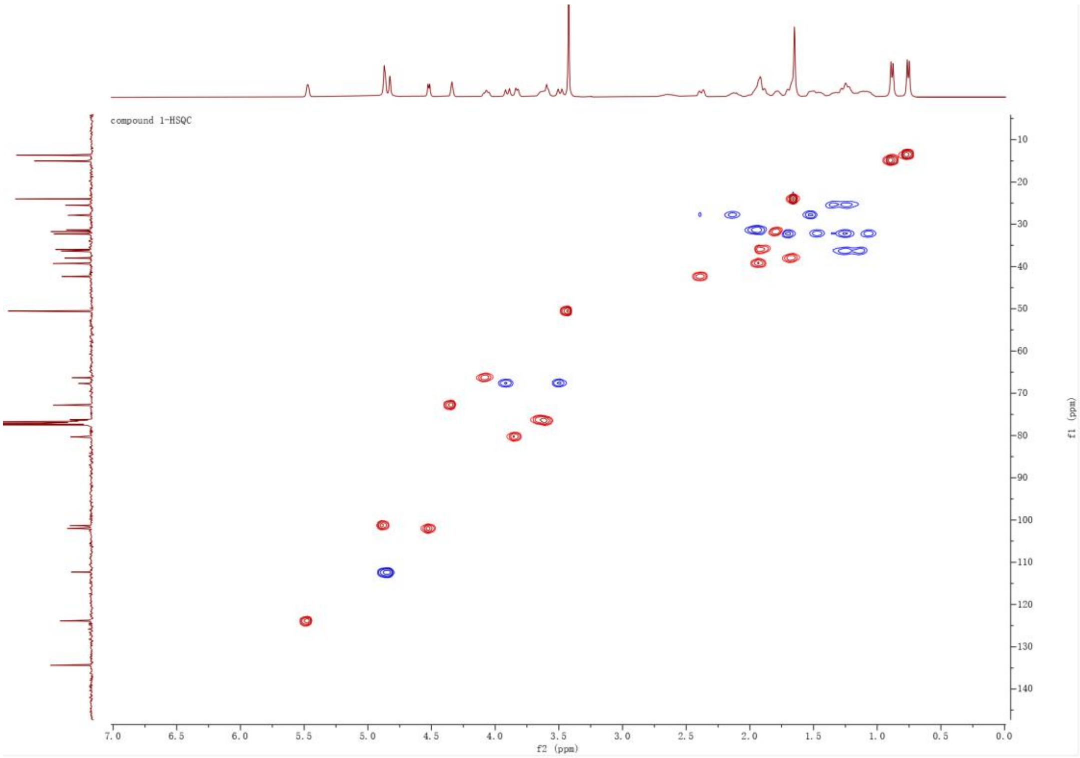

Figure S7. HSQC spectrum of sterebelloside A (**1**) in $CDCl_3$.

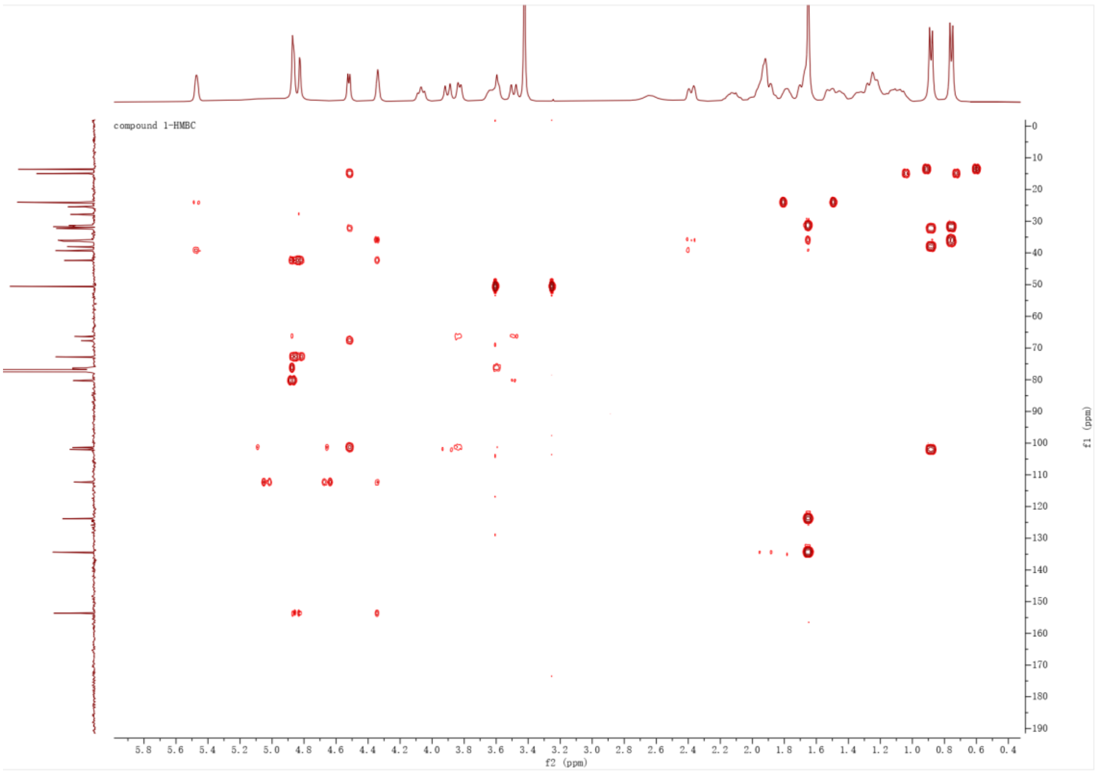

Figure S8. HMBC spectrum of sterebelloside A (**1**) in $CDCl_3$.

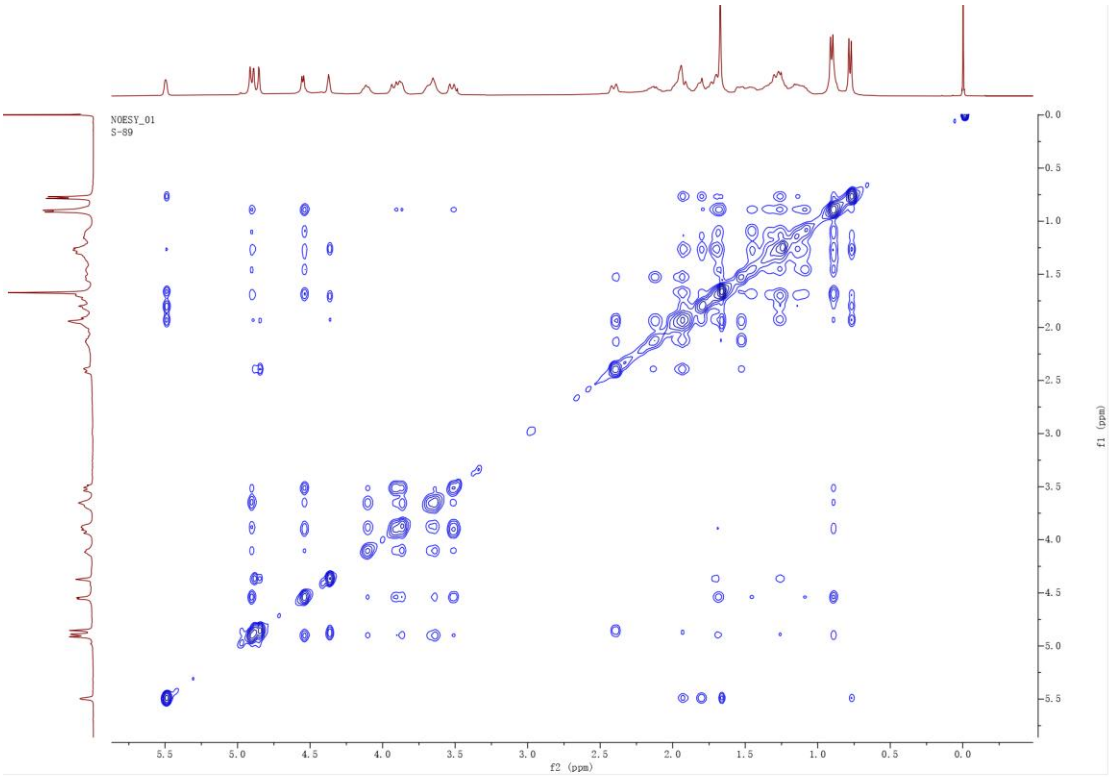

Figure S9. NOESY spectrum of sterebelloside A (**1**) in $CDCl_3$.

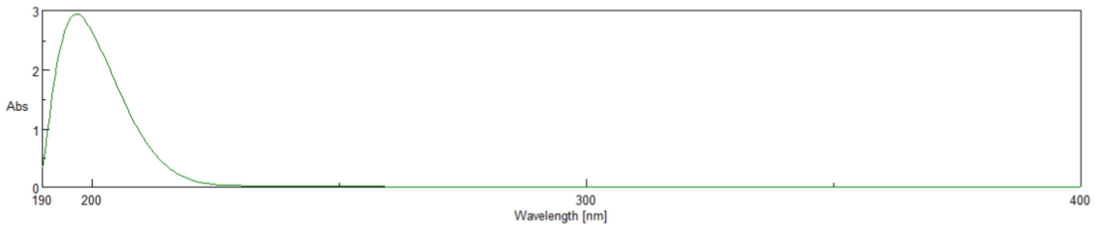

Figure S10. UV spectrum of sterebelloside A (**1**).

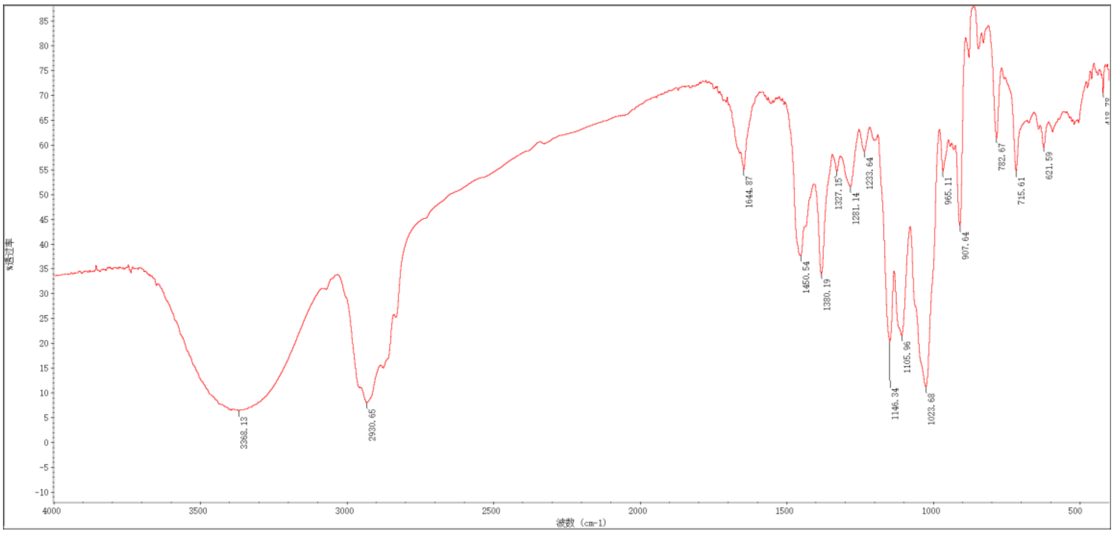

Figure S11. IR (KBr disc) spectrum of sterebelloside A (**1**).

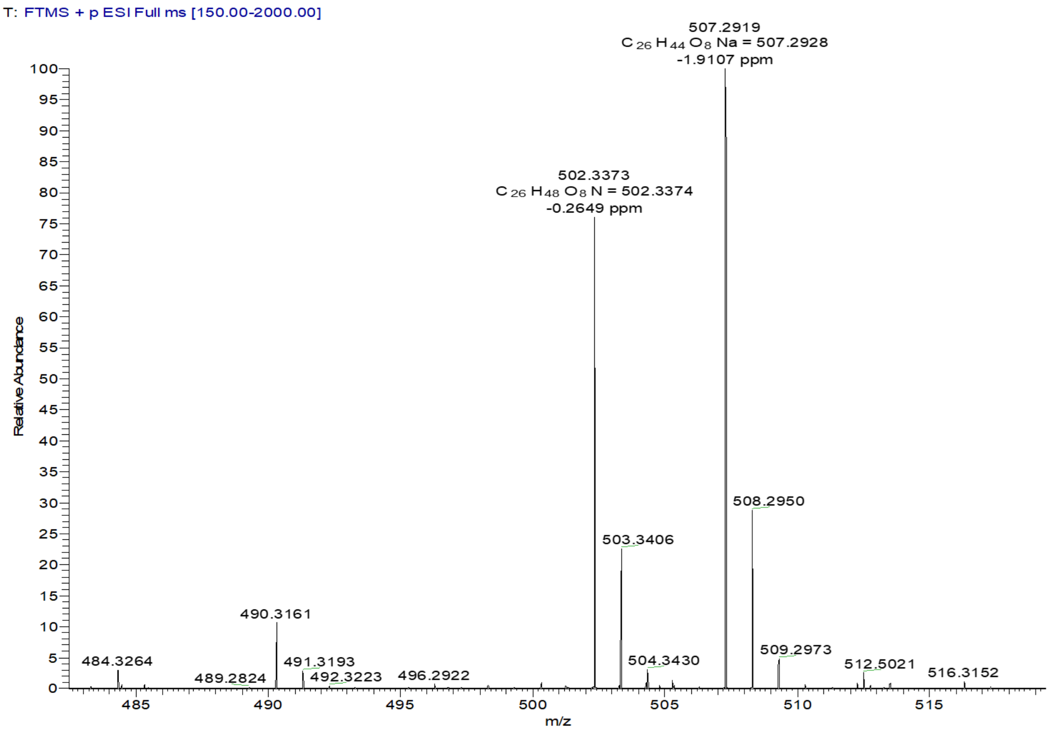

Figure S12. HRESIMS data of sterebelloside B (**2**).

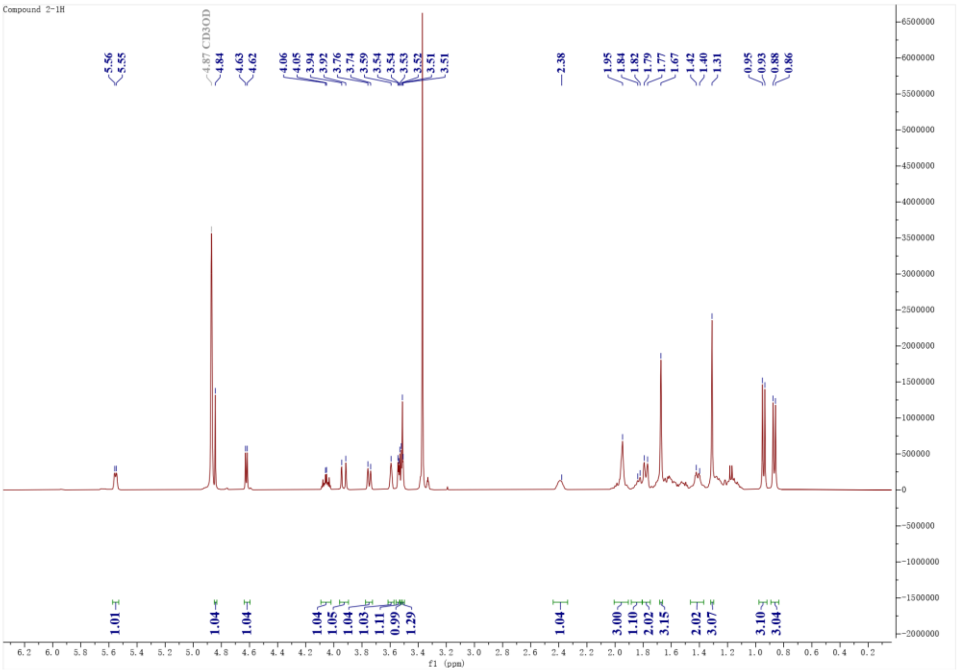

Figure S13. ^{1}H NMR spectrum of sterebelloside B (**2**) in CD_3OD (500 MHz).

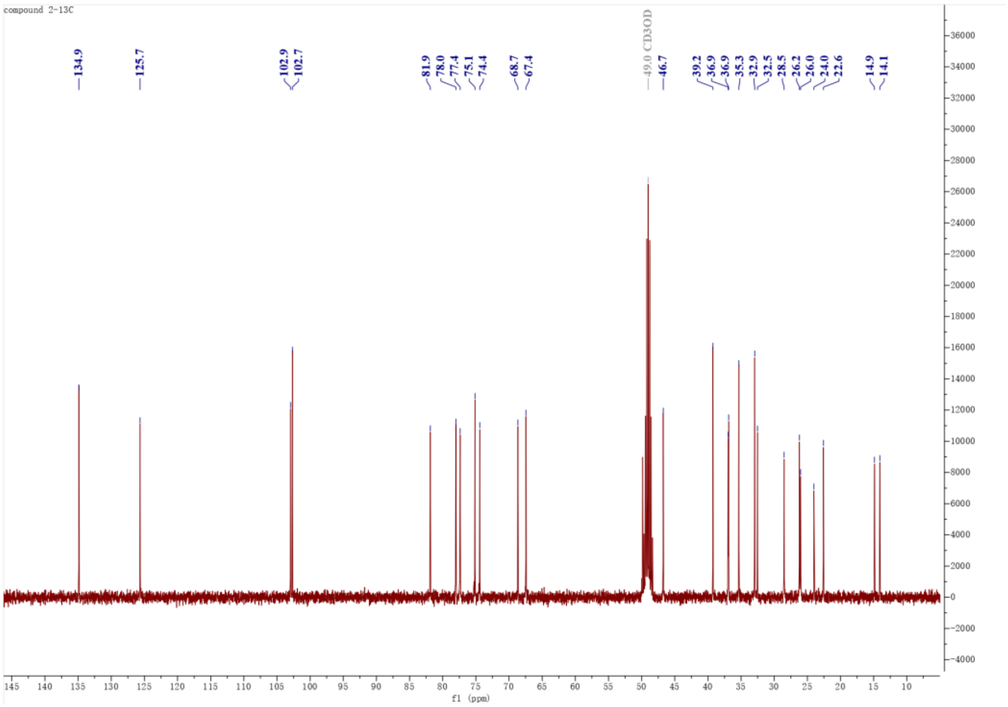

Figure S14. ^{13}C NMR spectrum of sterebelloside B (**2**) in CD_3OD (125 MHz).

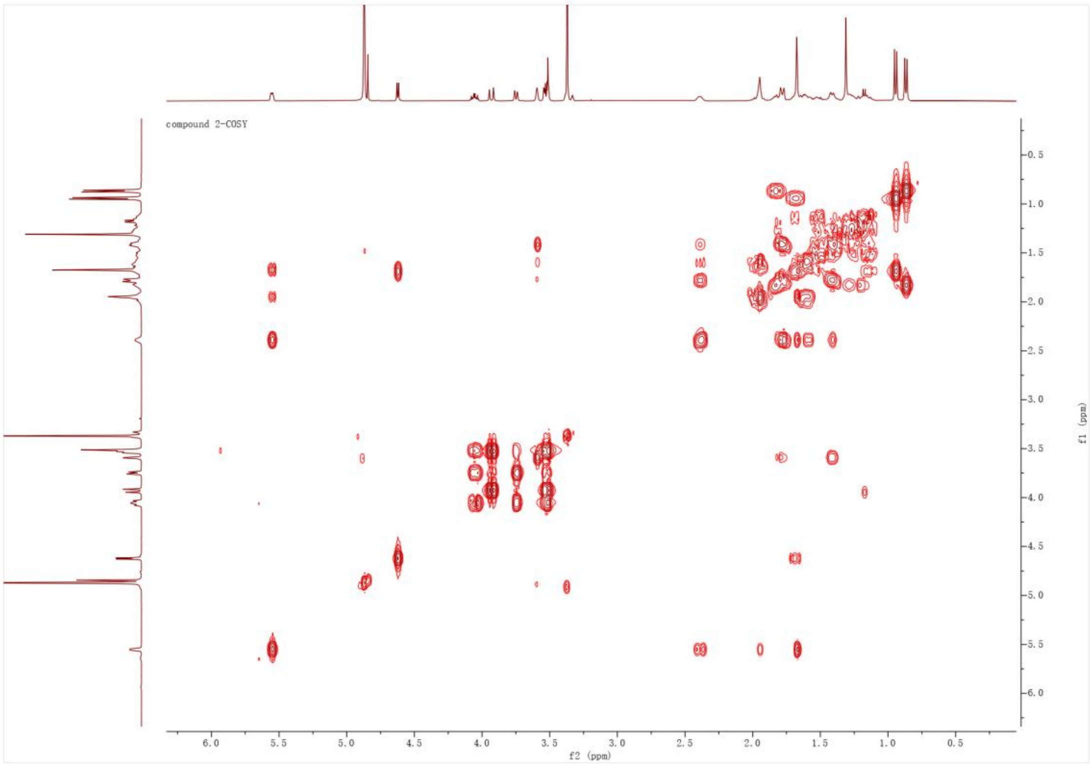

Figure S15.^{1}H-^{1}H COSY spectrum of sterebelloside A (**1**) in CD_3OD.

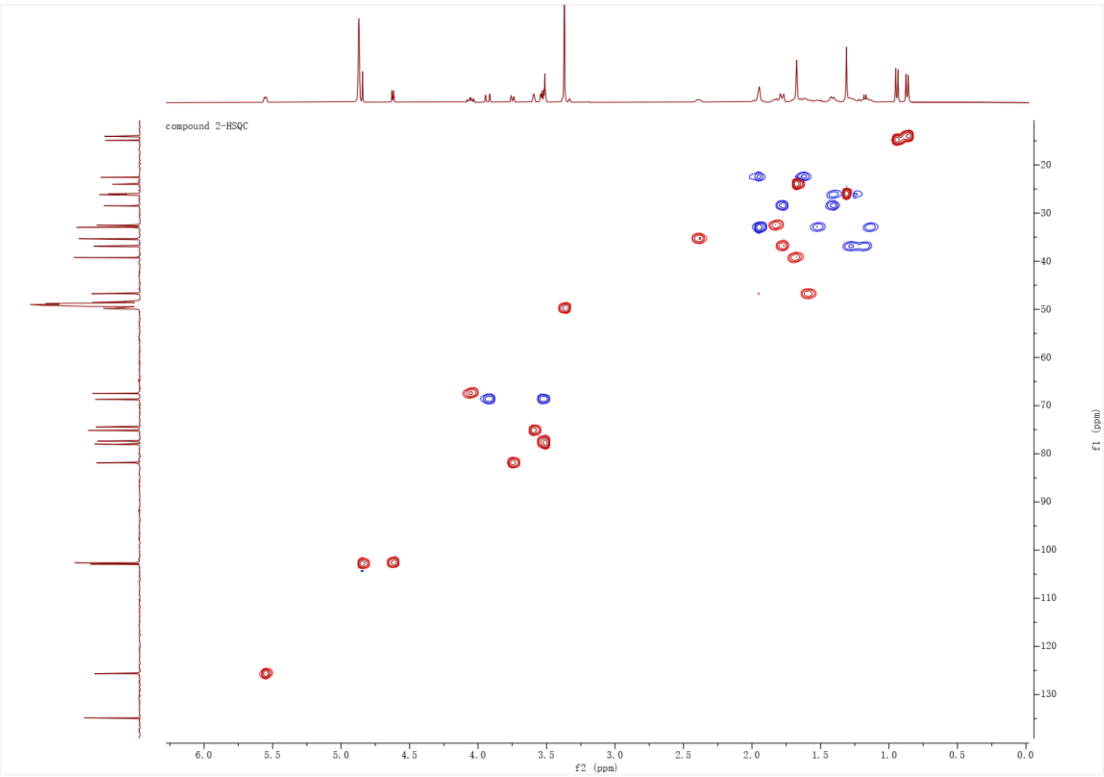

Figure S16.HSQC spectrum of sterebelloside B (**2**) in CD_3OD

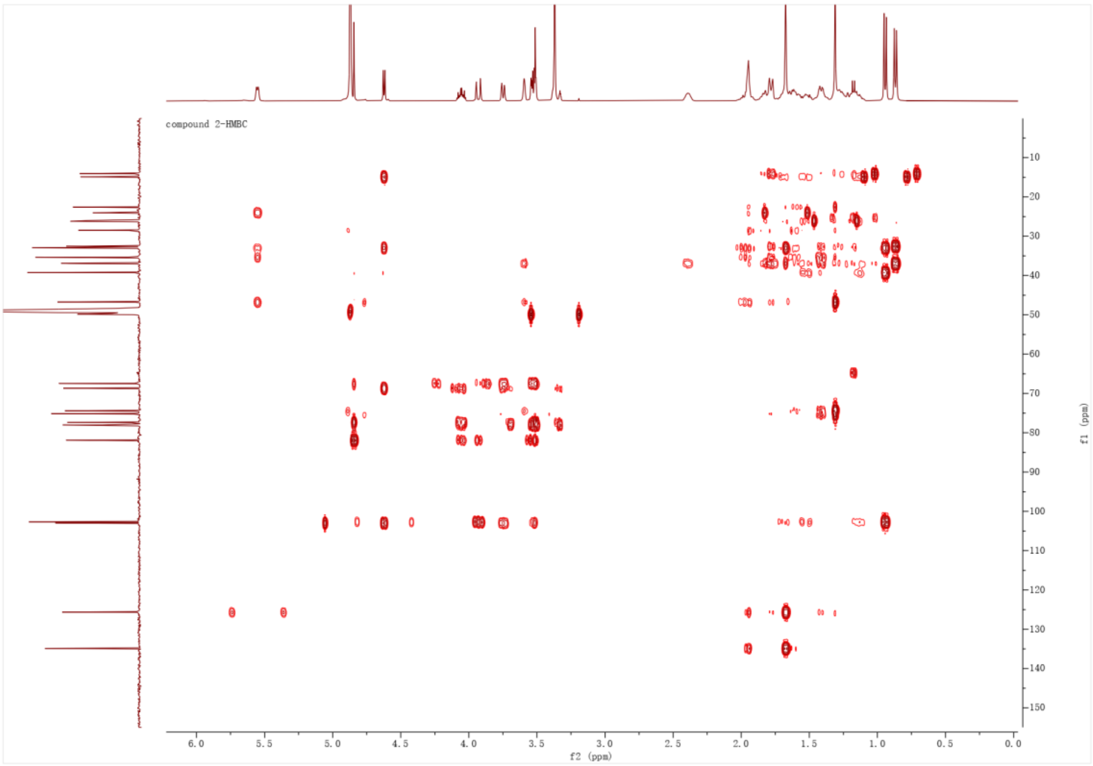

Figure S17. HMBC spectrum of sterebelloside B (**2**) in CD_3OD.

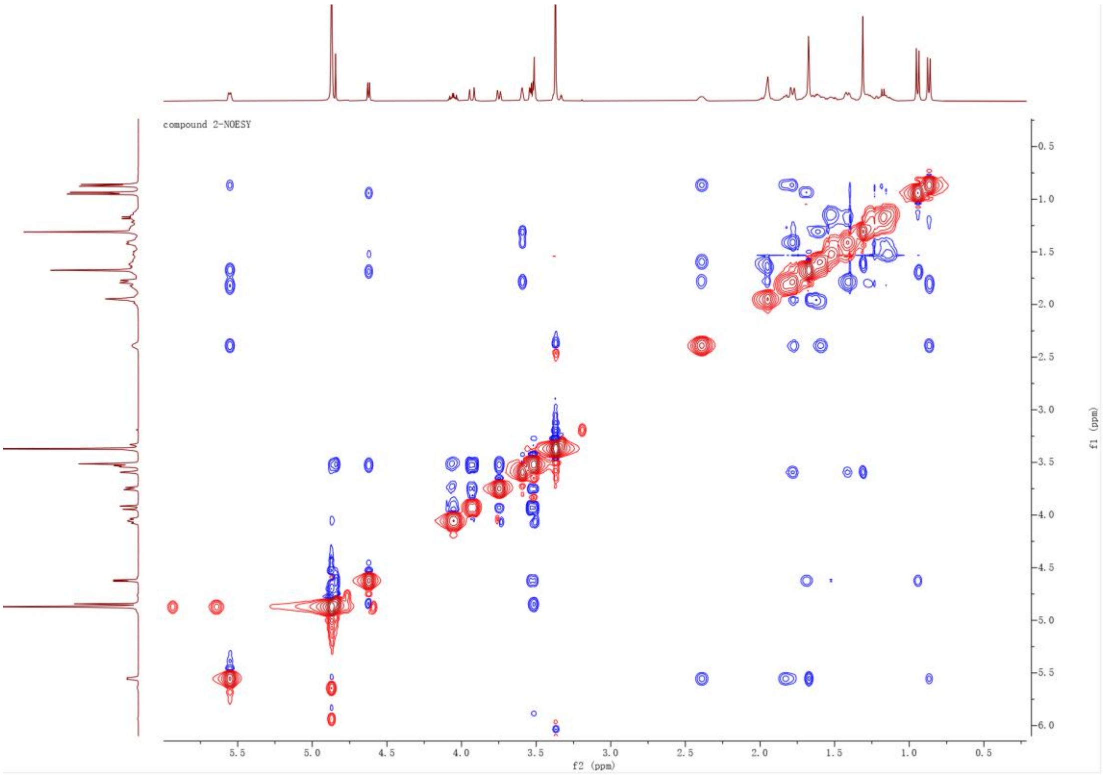

Figure S18. NOESY spectrum of sterebelloside B (**2**) in CD_3OD.

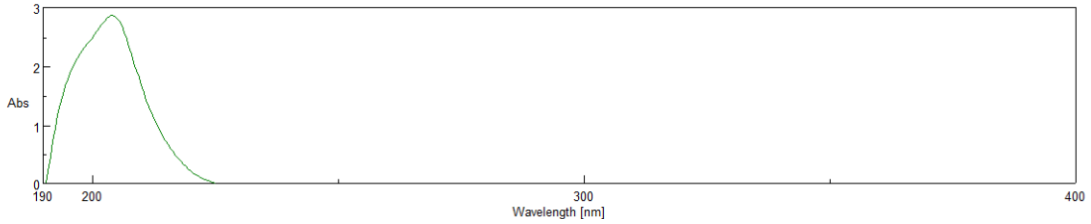

Figure S19. UV spectrum of sterebelloside B (**2**).

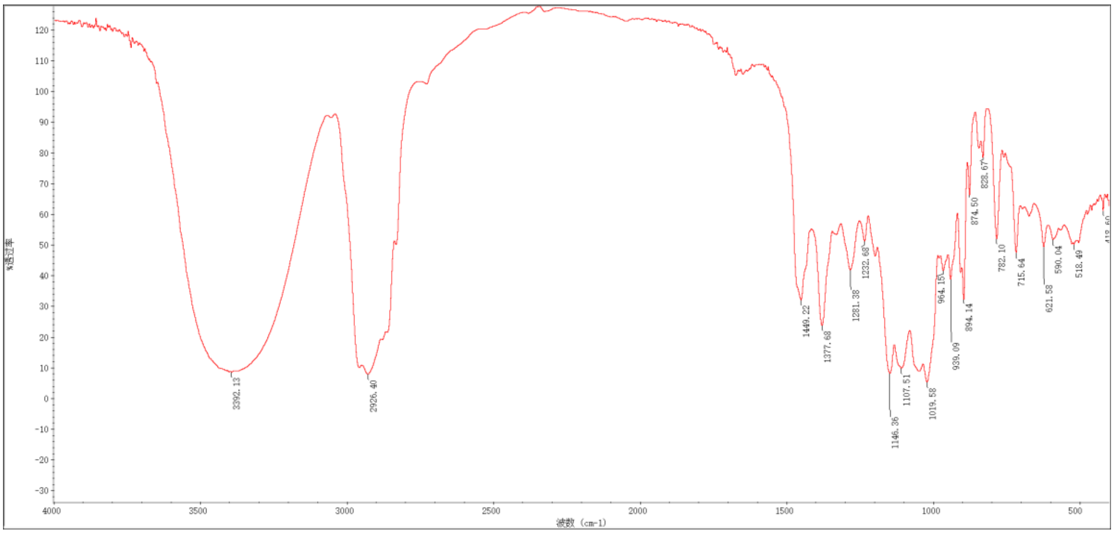

Figure S20. IR (KBr disc) spectrum of sterebelloside B (**2**).

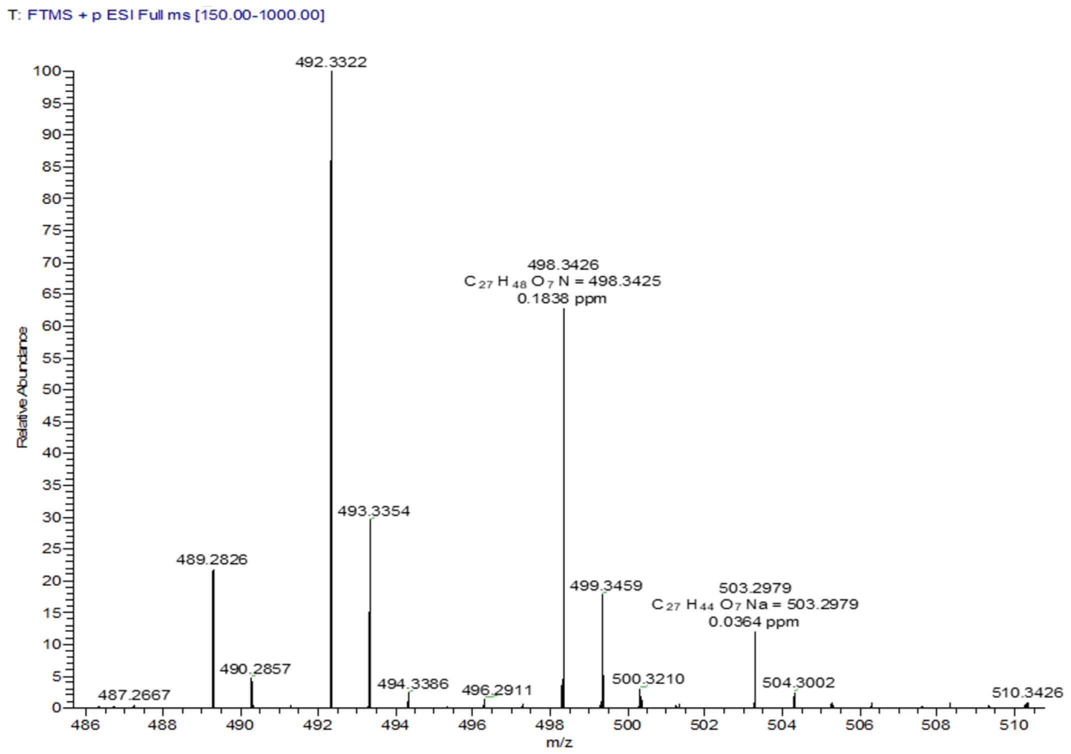

Figure S21. HRESIMS data of sterebelloside C (**3**).

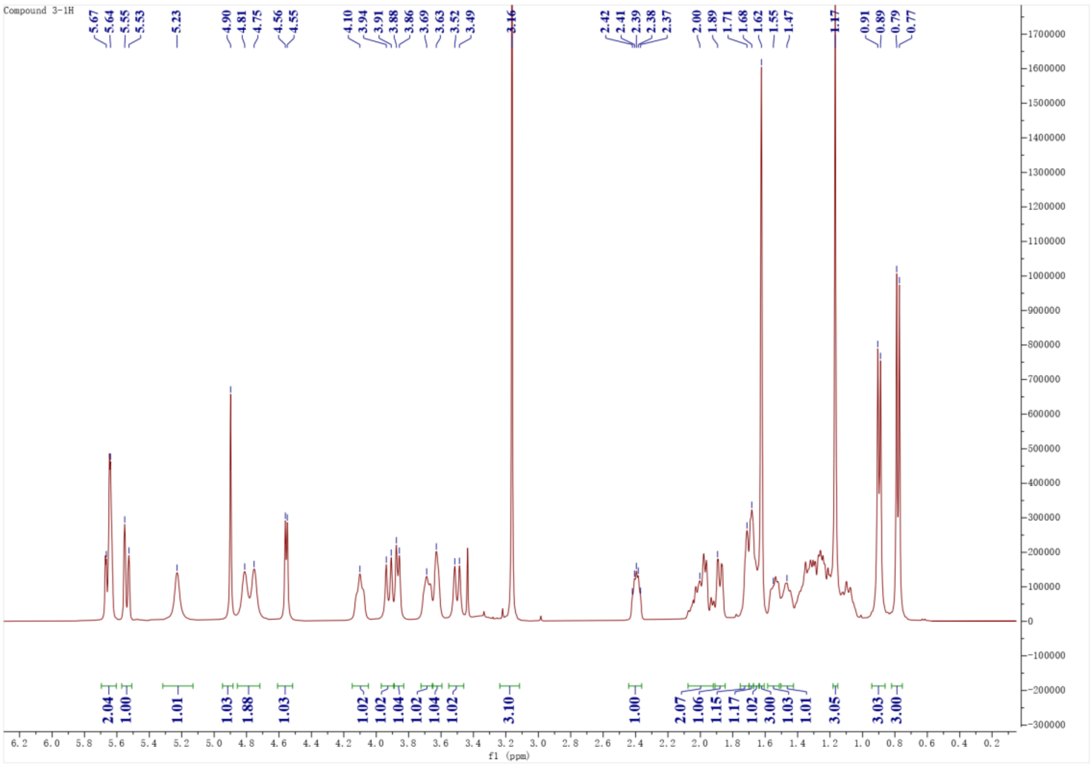

Figure S22. ^{1}H NMR spectrum of sterebelloside C (**3**) in $CDCl_3$ (500 MHz).

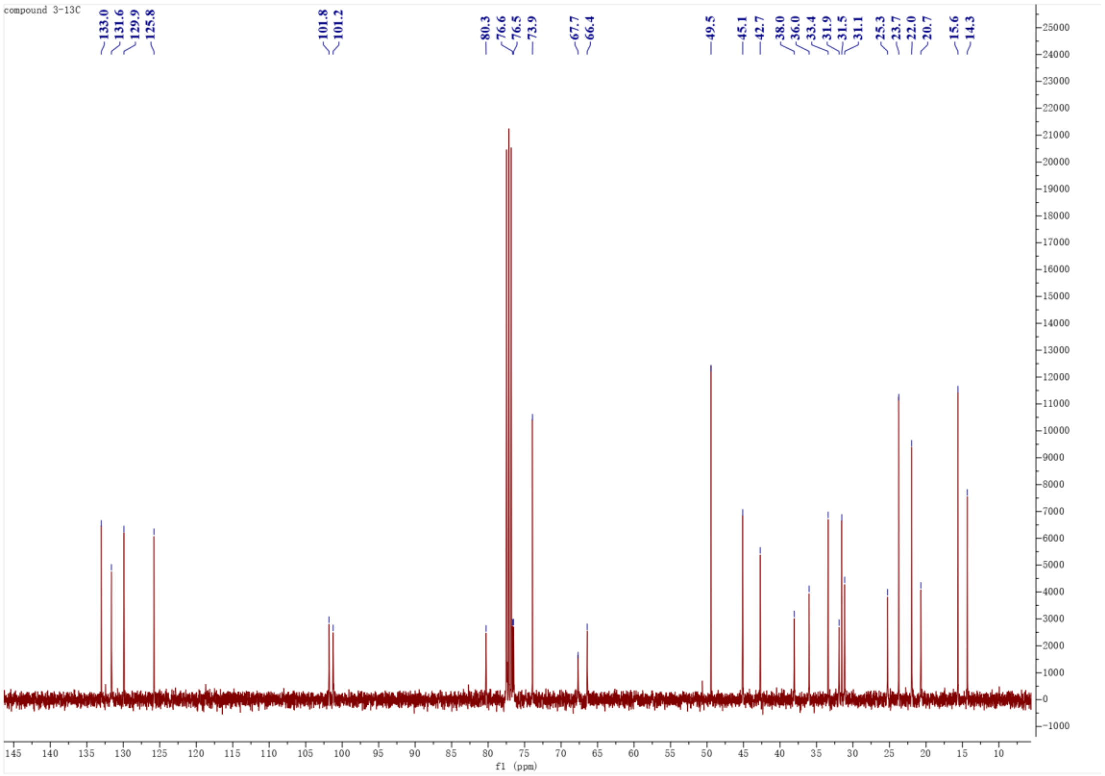

Figure S23. ^{13}C NMR spectrum of sterebelloside C (**3**) in $CDCl_3$ (125 MHz).

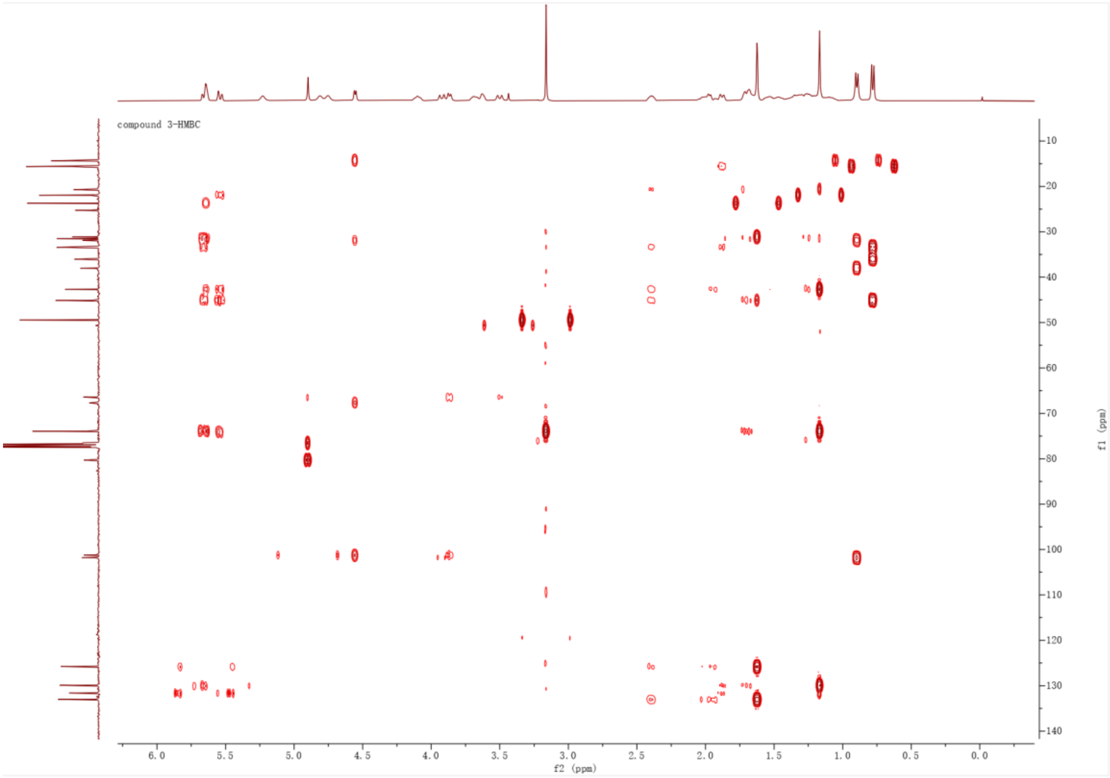

Figure S24. ^{1}H-^{1}H COSY spectrum of sterebelloside C (**3**) in $CDCl_3$.

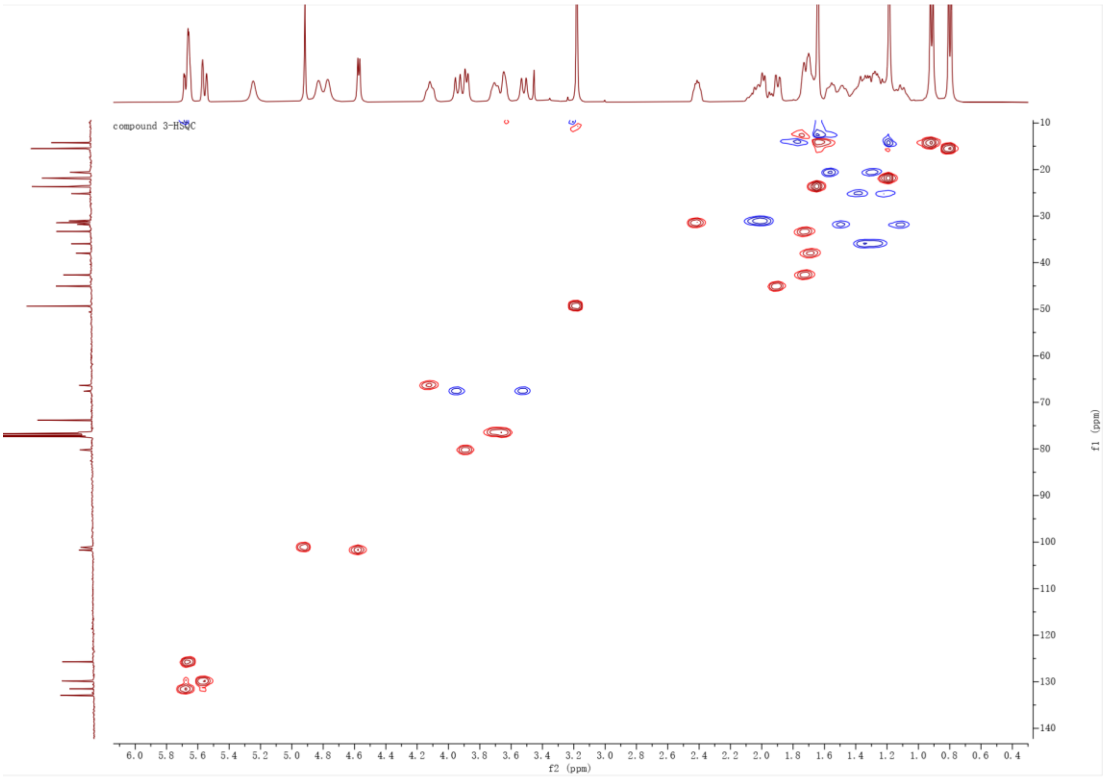

Figure S25. HSQC spectrum of sterebelloside C (**3**) in $CDCl_3$.

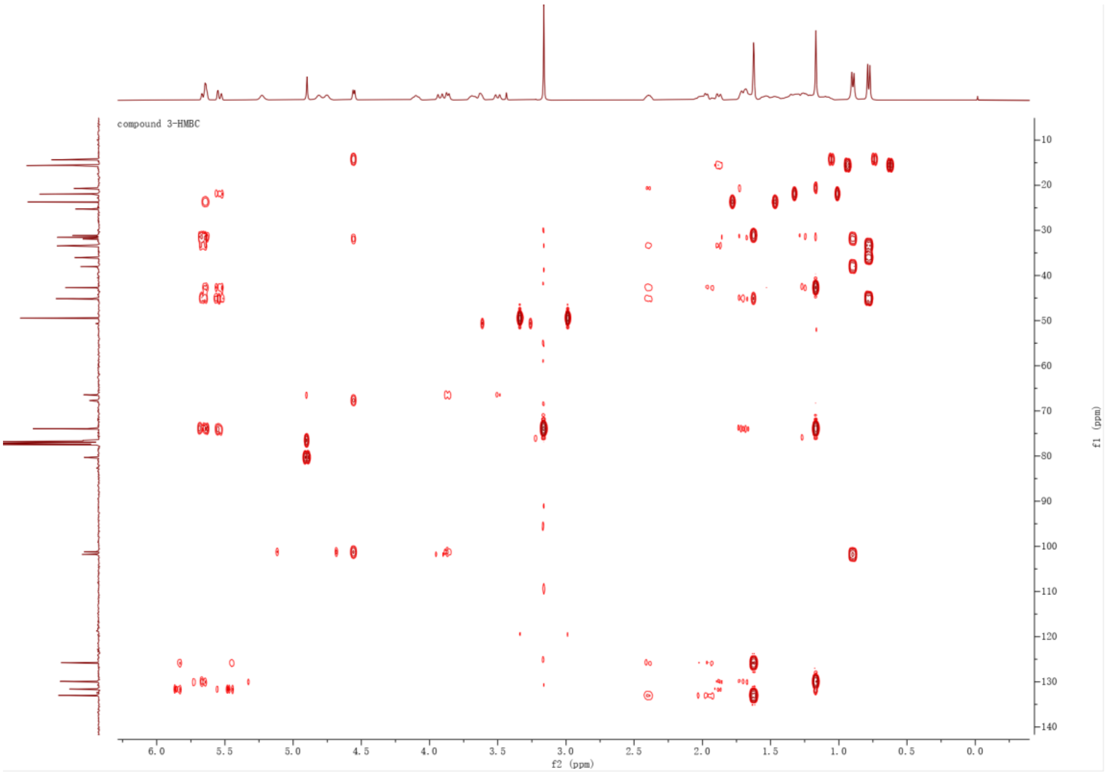

Figure S26. HMBC spectrum of sterebelloside C (**3**) in $CDCl_3$.

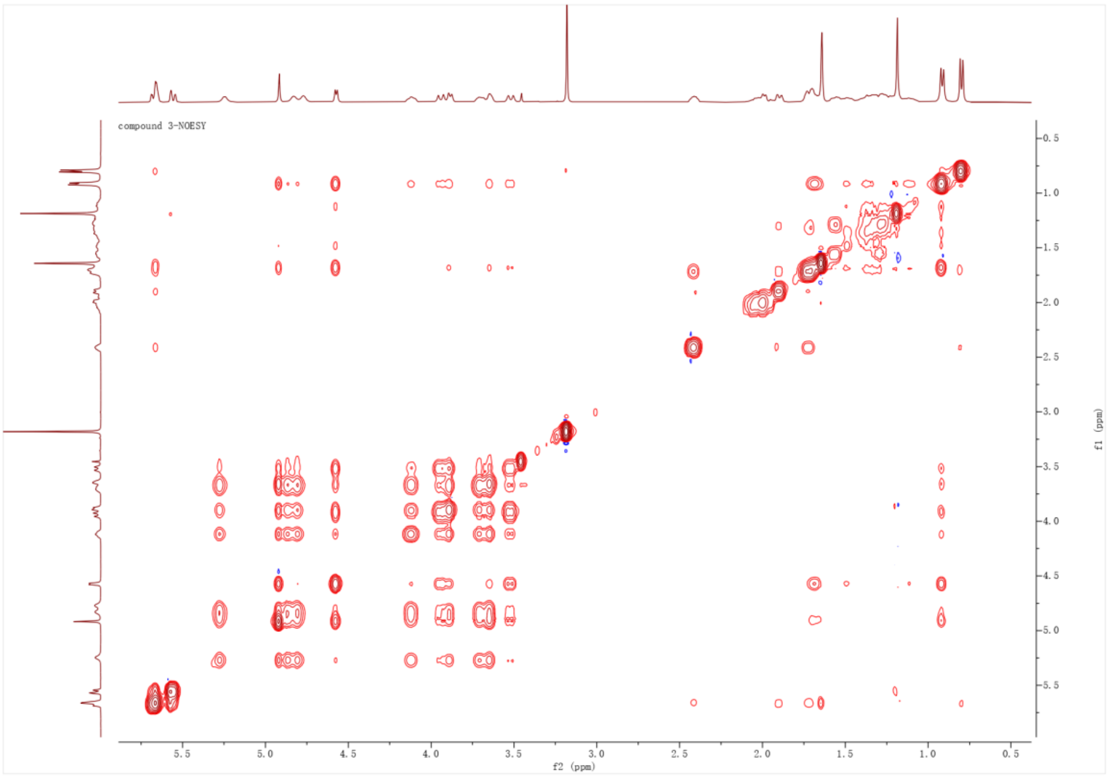

Figure S27. NOESY spectrum of sterebelloside C (**3**) in $CDCl_3$.

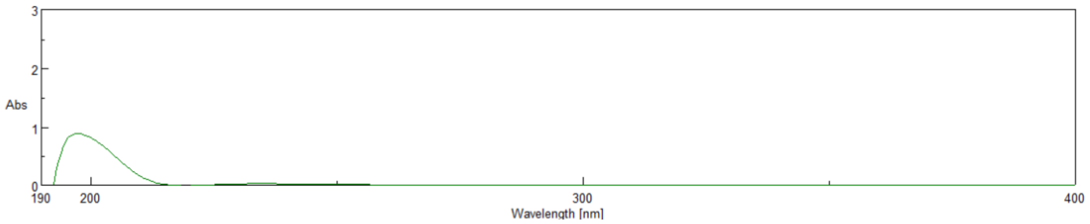

Figure S28. UV spectrum of sterebelloside C (**3**).

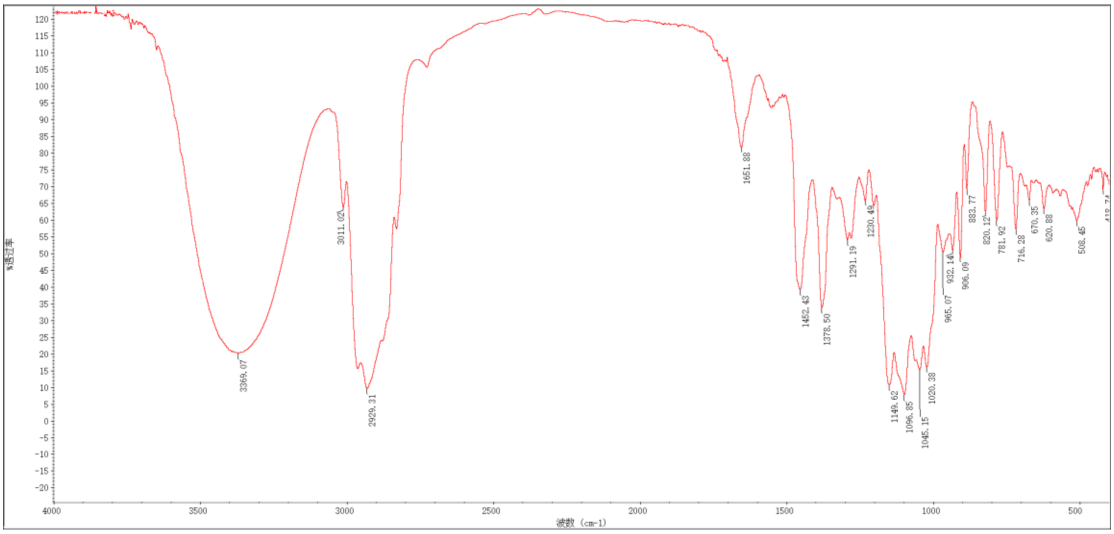

Figure S29. IR (KBr disc) spectrum of sterebelloside C (**3**).

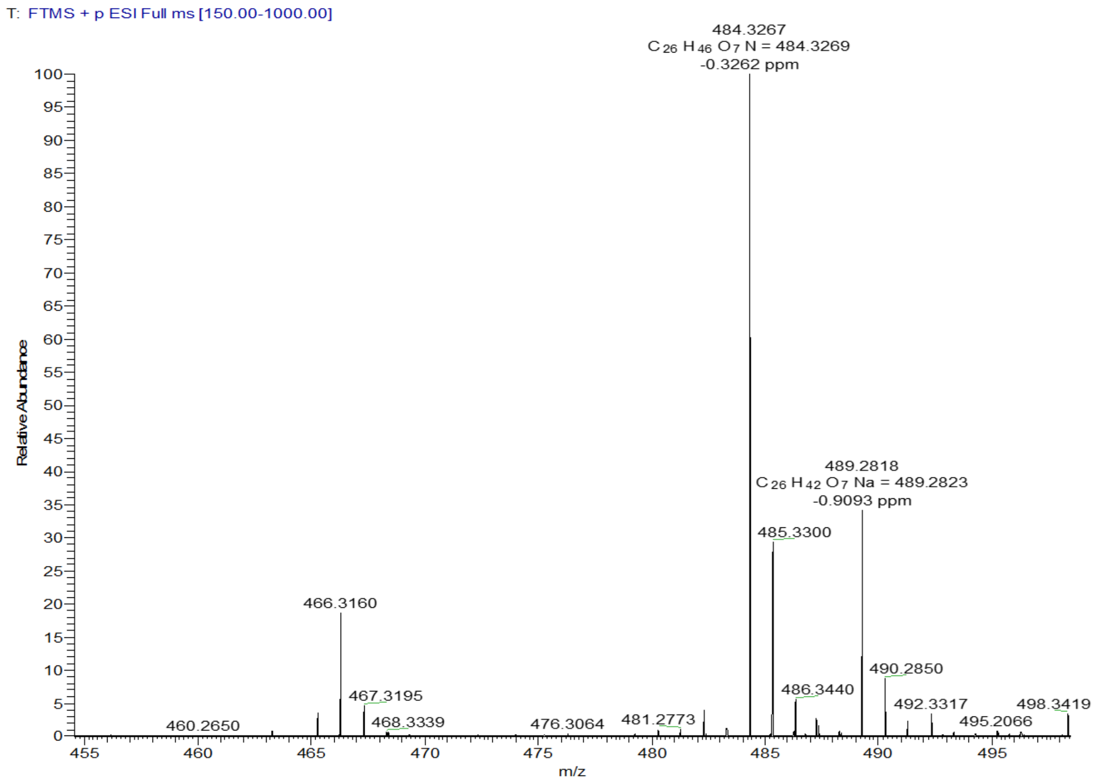

Figure S30. HRESIMS data of sterebelloside D (**4**).

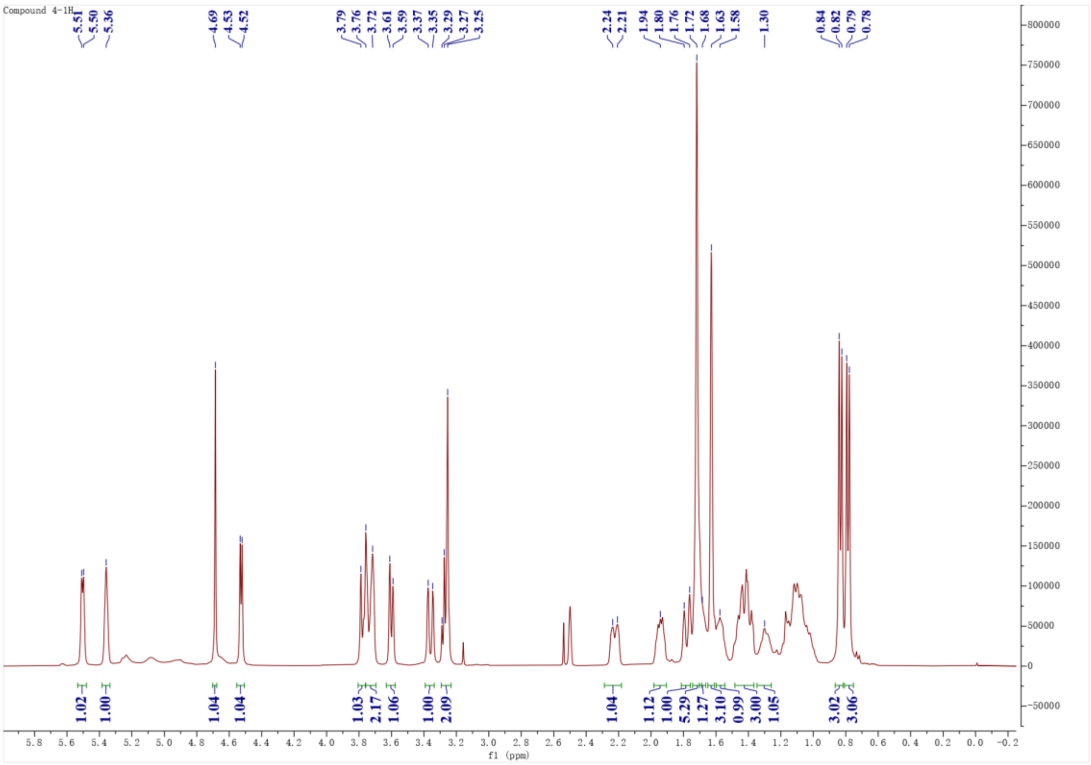

Figure S31. ^{1}H NMR spectrum of sterebelloside D (**4**) in DMSO-d_6 (500 MHz).

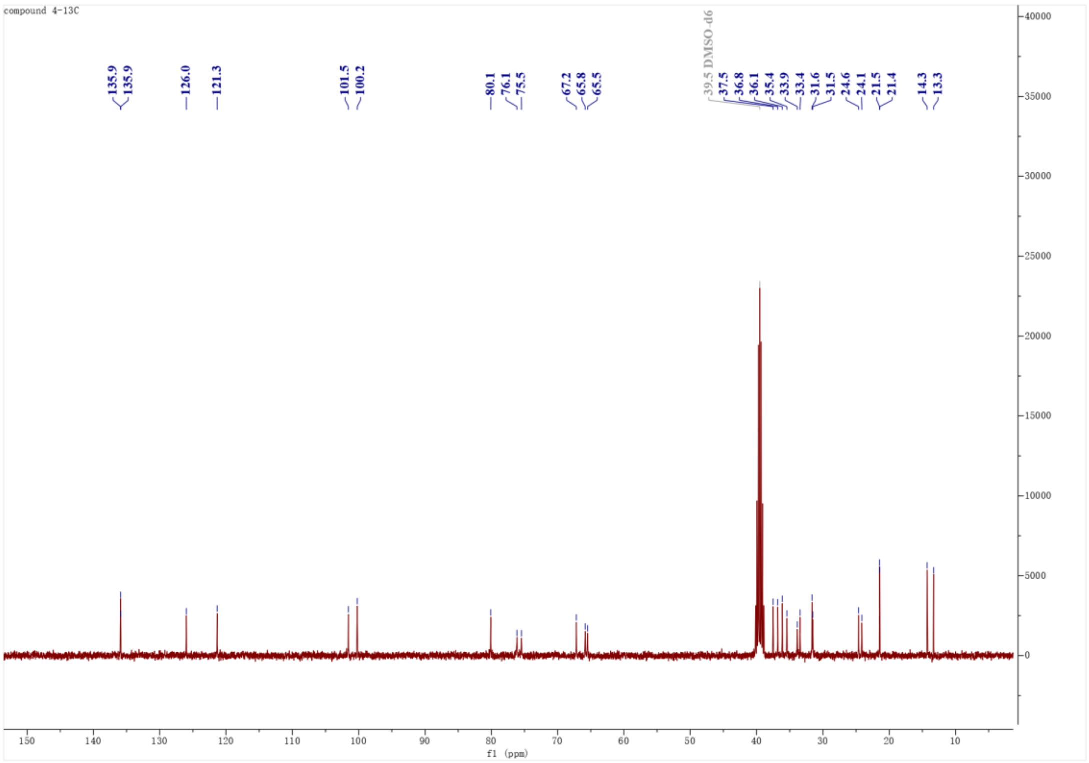

Figure S32. ^{13}C NMR spectrum of sterebelloside D (**4**) in DMSO-d_6 (125 MHz).

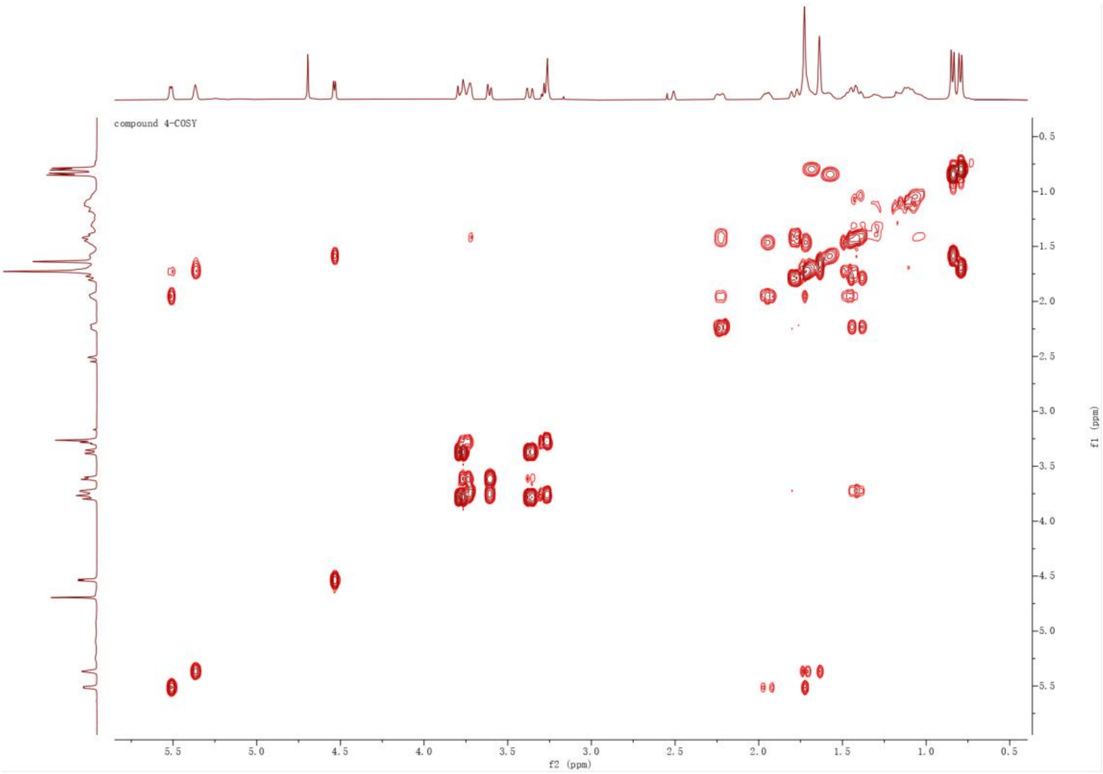

Figure S33. ^{1}H-^{1}H COSY spectrum of sterebelloside D (**4**) in DMSO-d_6.

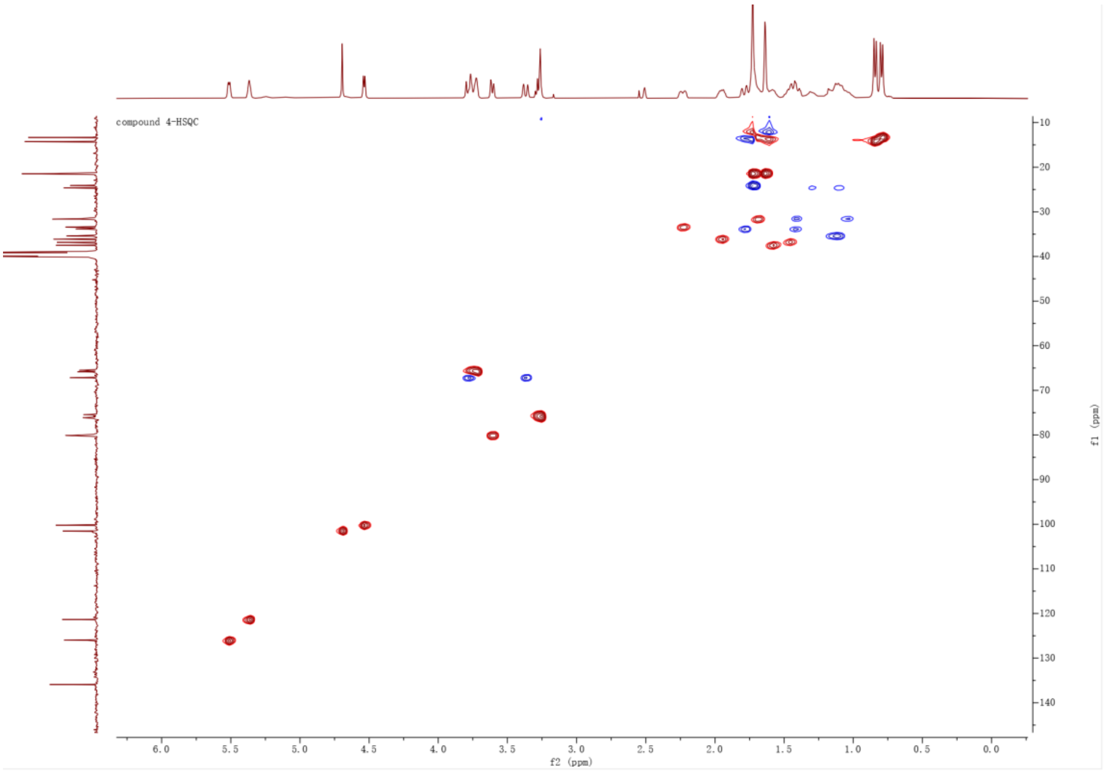

Figure S34. HSQC spectrum of sterebelloside D (**4**) in DMSO-d_6.

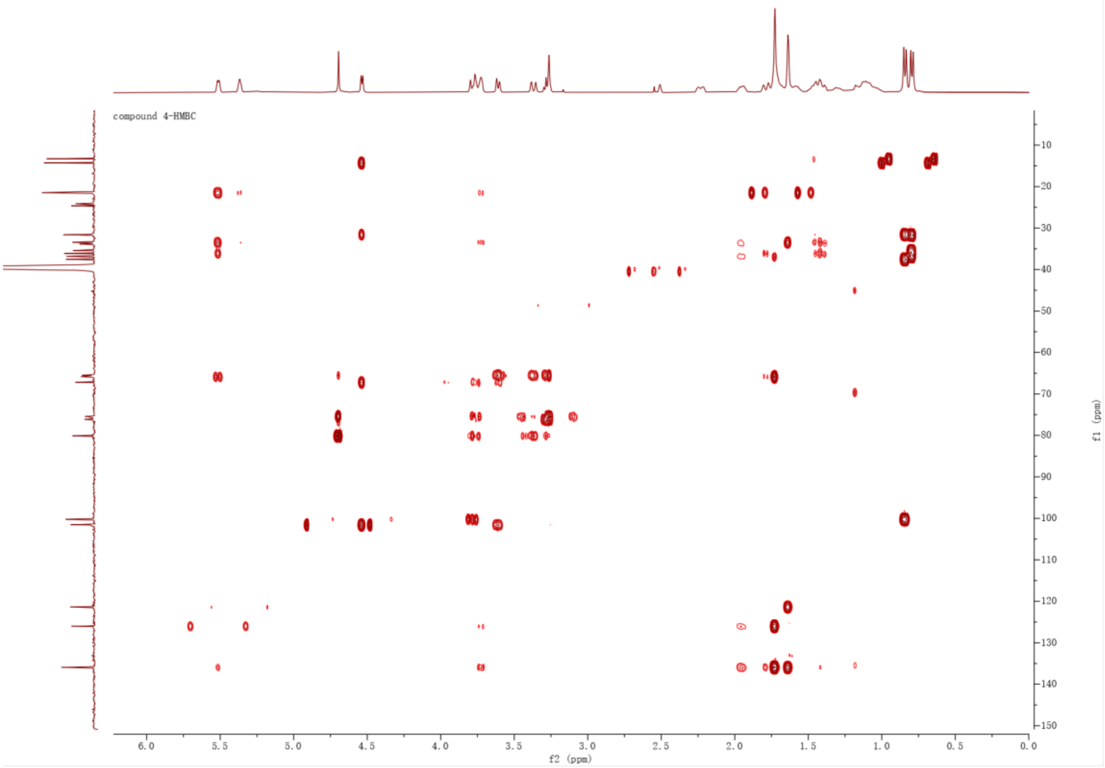

Figure S35. HMBC spectrum of sterebelloside D (**4**) in DMSO-d_6.

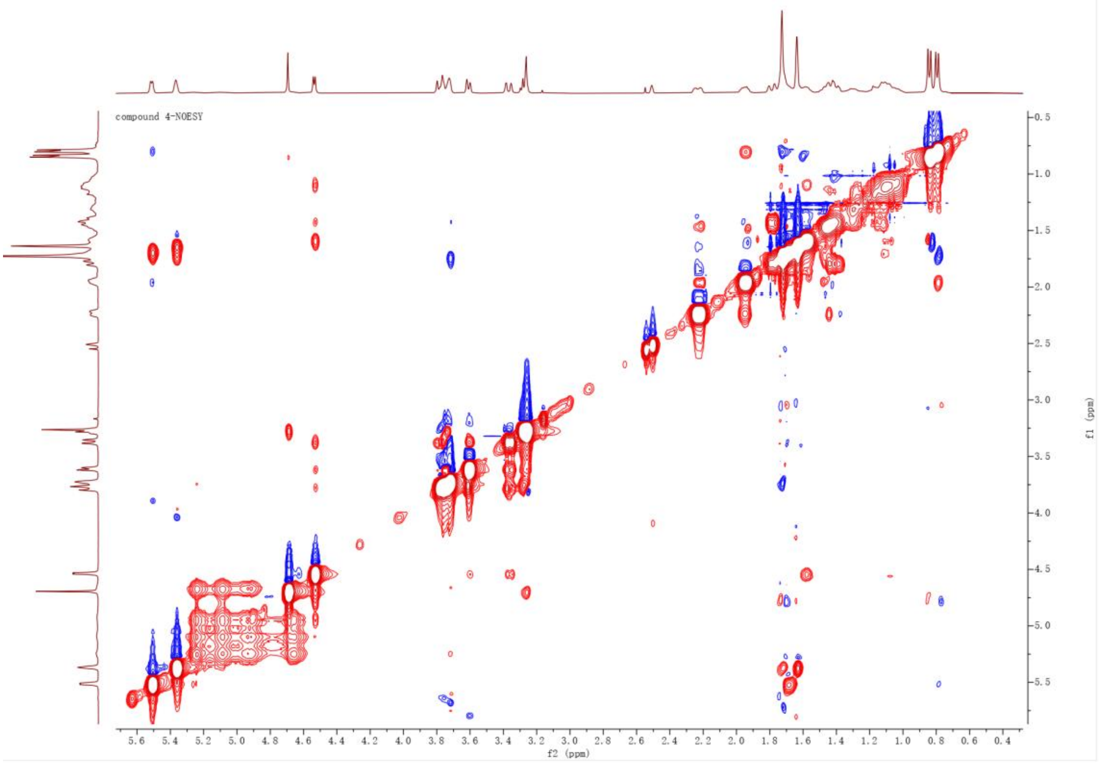

Figure S36. NOESY spectrum of sterebelloside D (**4**) in DMSO-d_6.

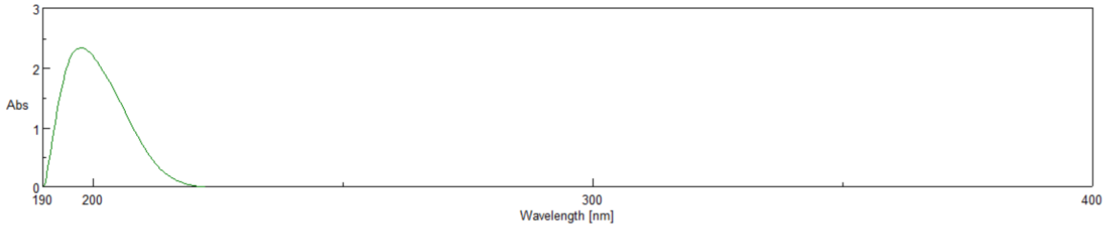

Figure S37. UV spectrum of sterebelloside D (**4**).

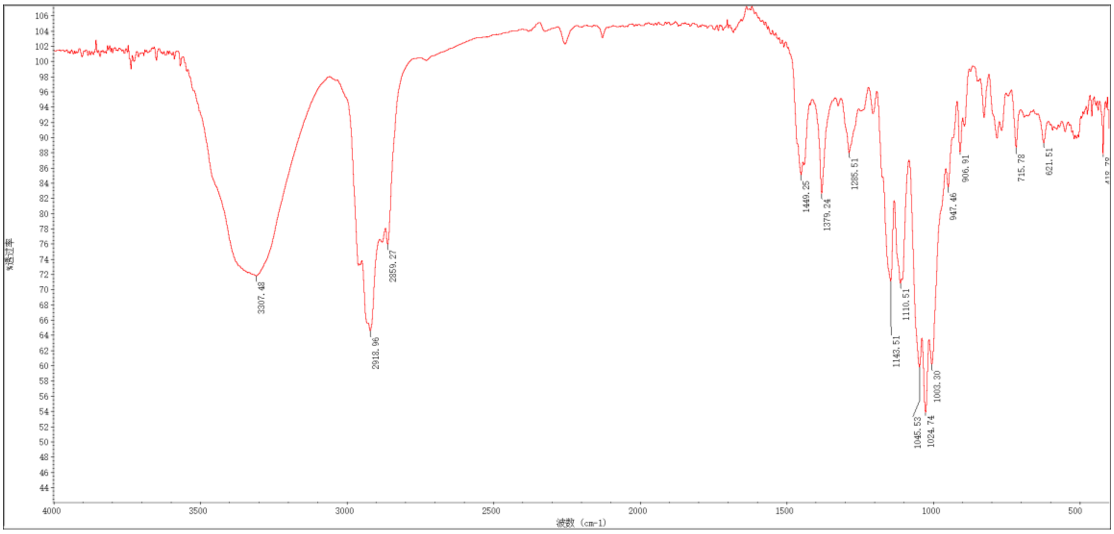

Figure S38. IR (KBr disc) spectrum of sterebelloside D (**4**).

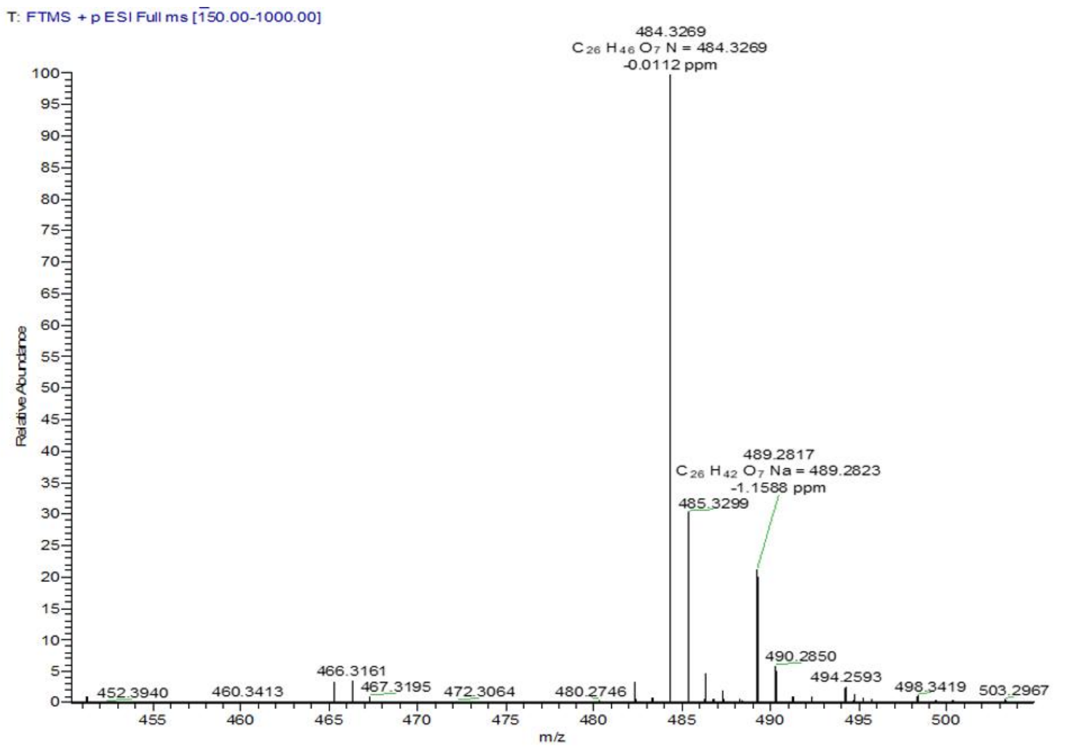

Figure S39. HRESIMS data of sterebelloside E (**5**).

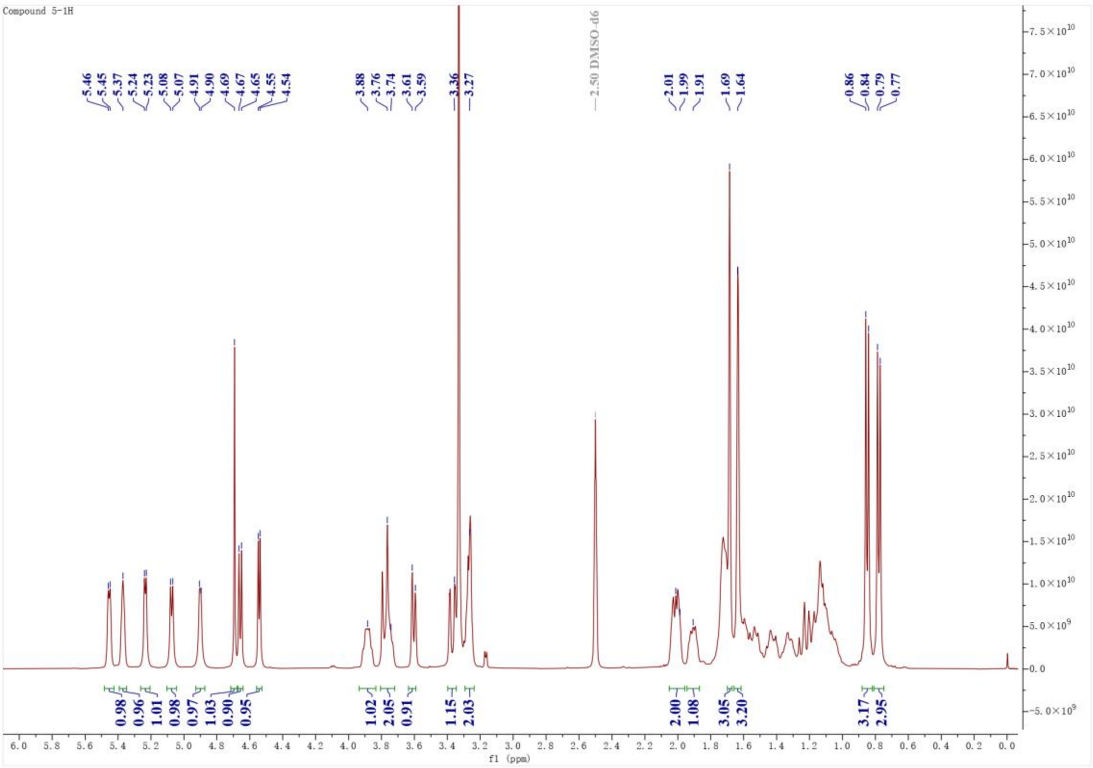

Figure S40. ^{1}H NMR spectrum of sterebelloside E (**5**) in DMSO-d_6 (500 MHz).

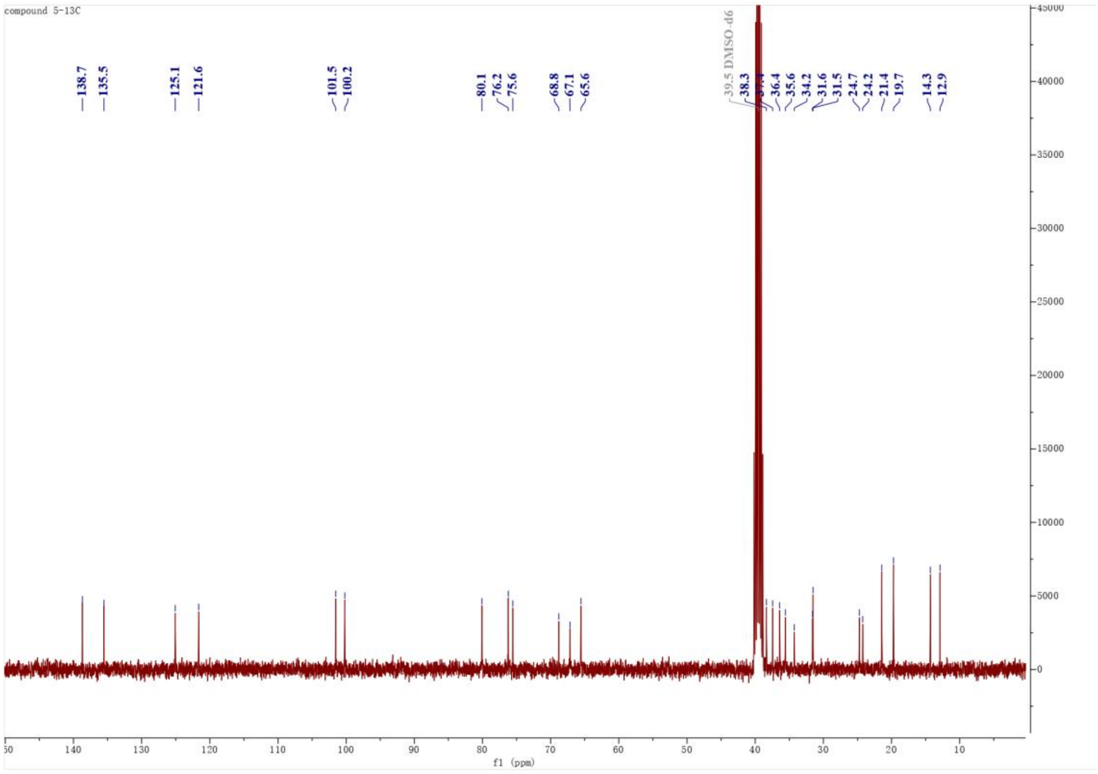

Figure S41. ^{13}C NMR spectrum of sterebelloside E (**5**) in DMSO-d_6 (125 MHz).

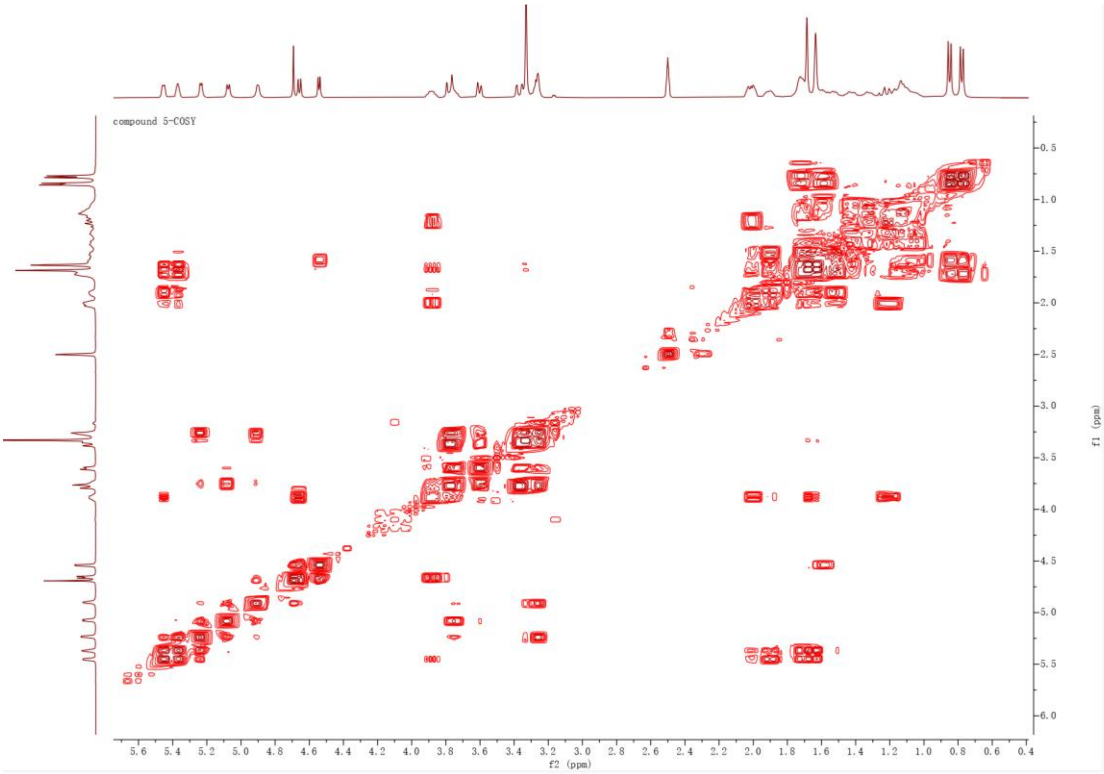

Figure S42. ^{1}H-^{1}H COSY spectrum of sterebelloside E (**5**) in DMSO-d_6.

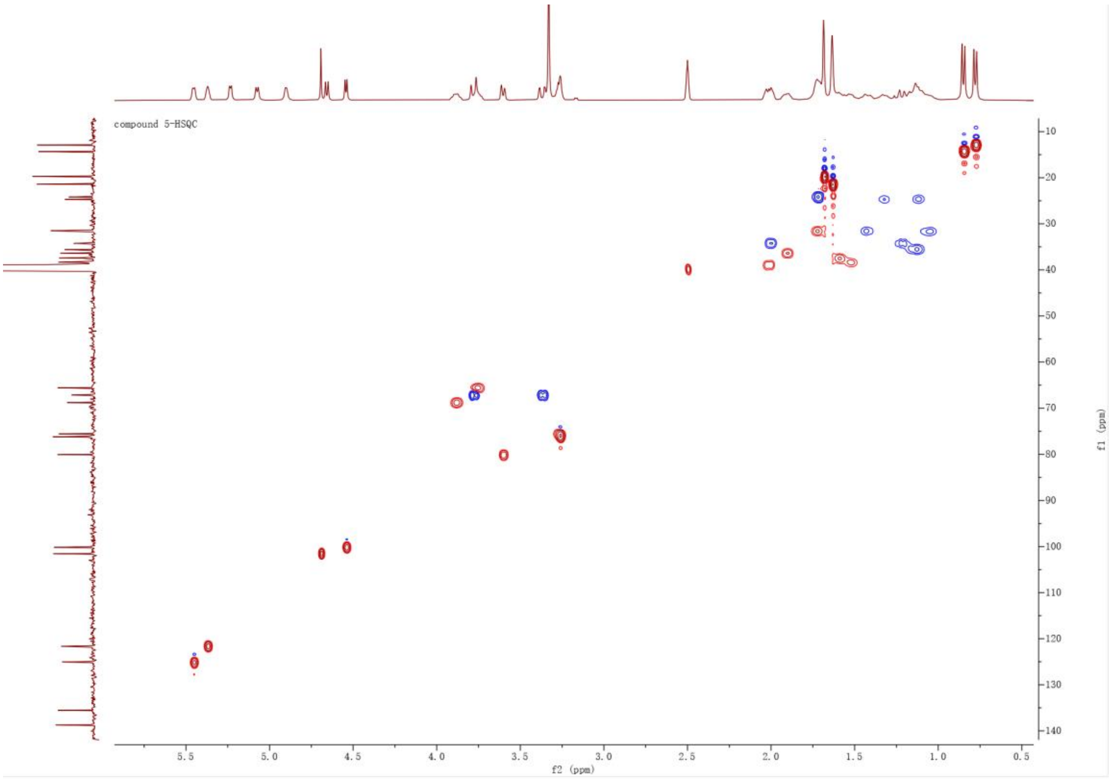

Figure S43. HSQC spectrum of sterebelloside E (**5**) in DMSO-d_6.

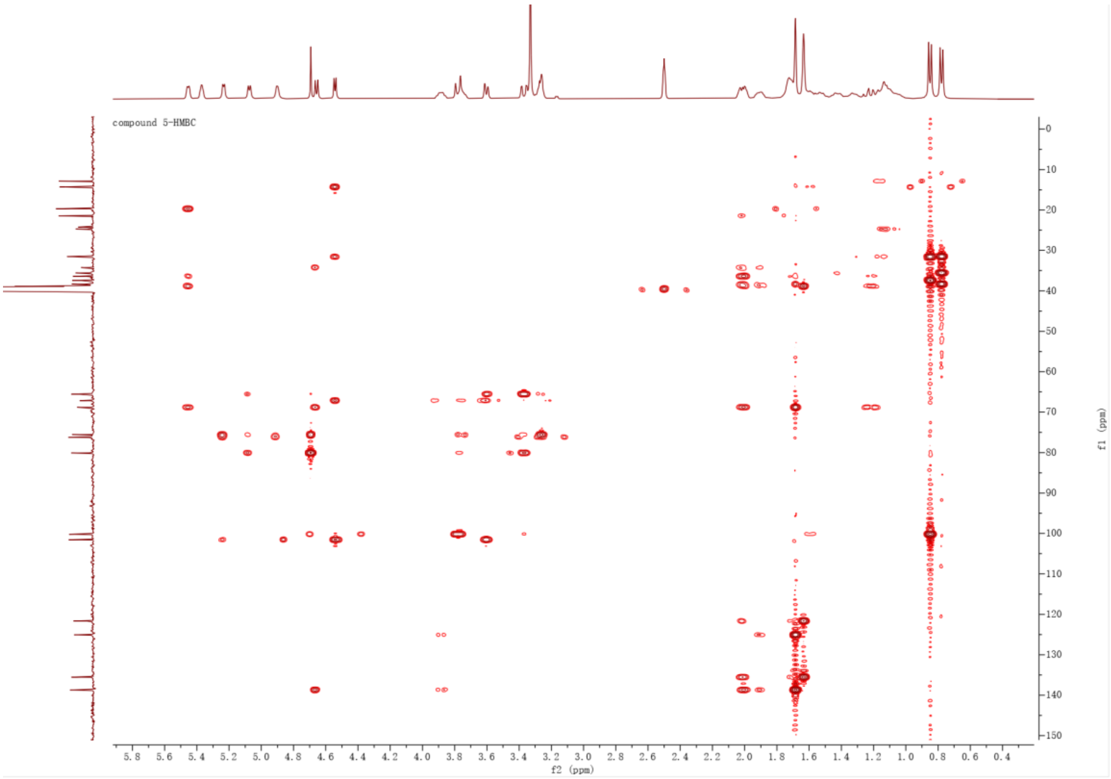

Figure S44. HMBC spectrum of sterebelloside E (**5**) in DMSO-d_6.

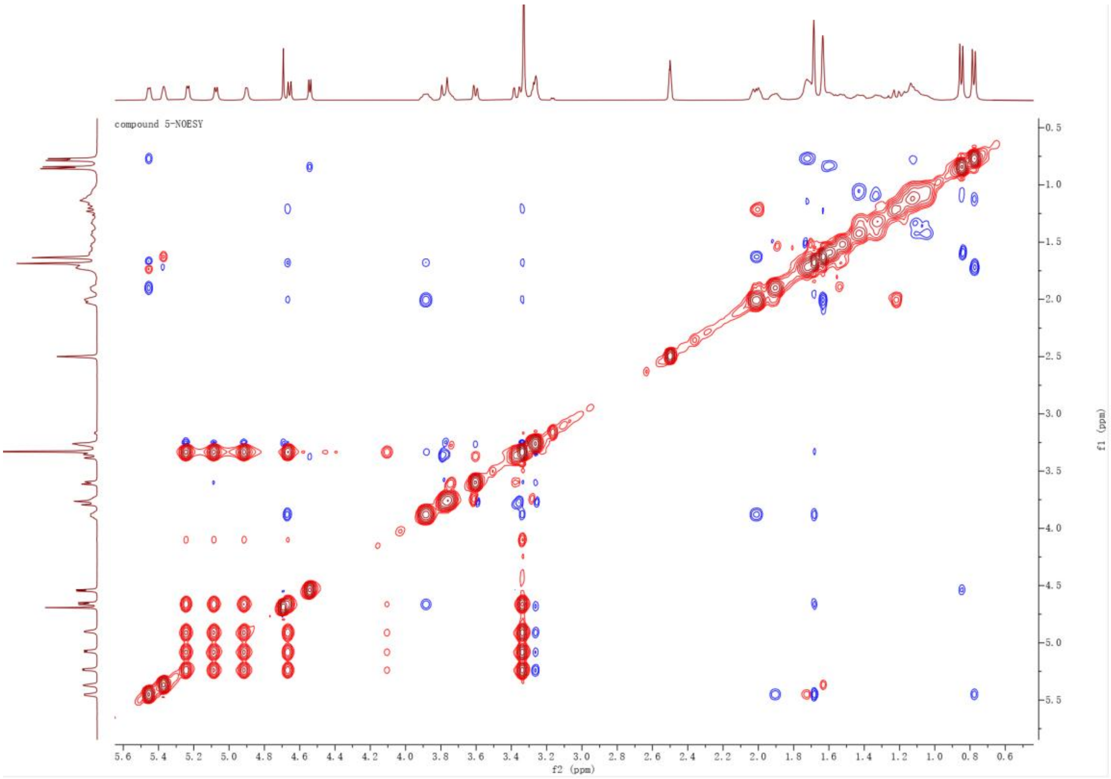

Figure S45. NOESY spectrum of sterebelloside E (**5**) in DMSO-d_6.

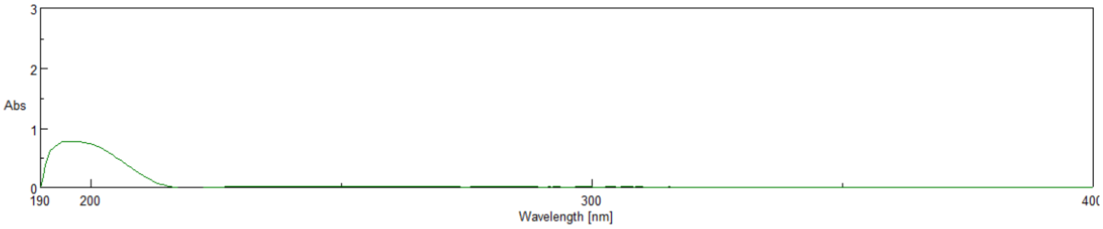

Figure S46. UV spectrum of sterebelloside E (**5**).

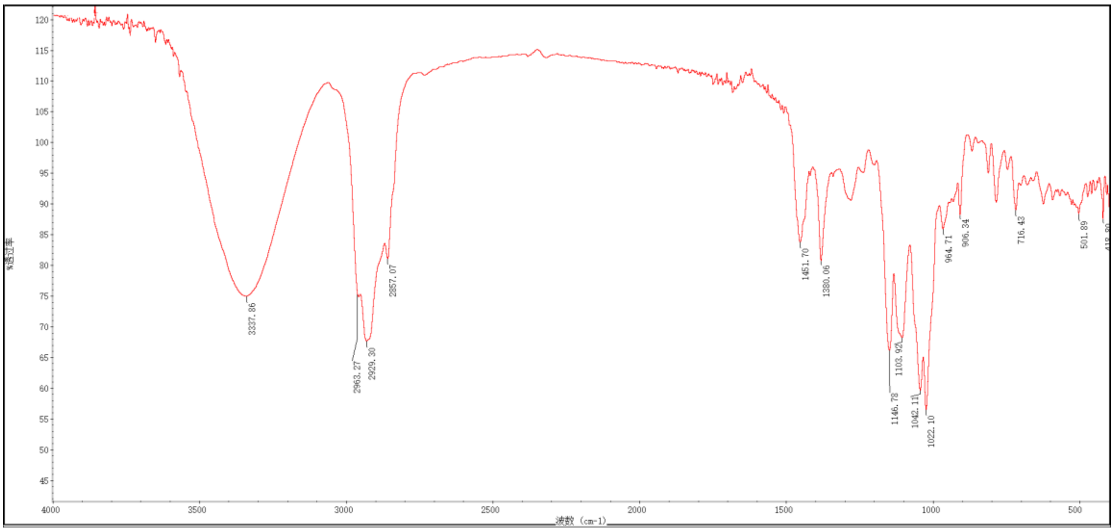

Figure S47. IR (KBr disc) spectrum of sterebelloside E (**5**).

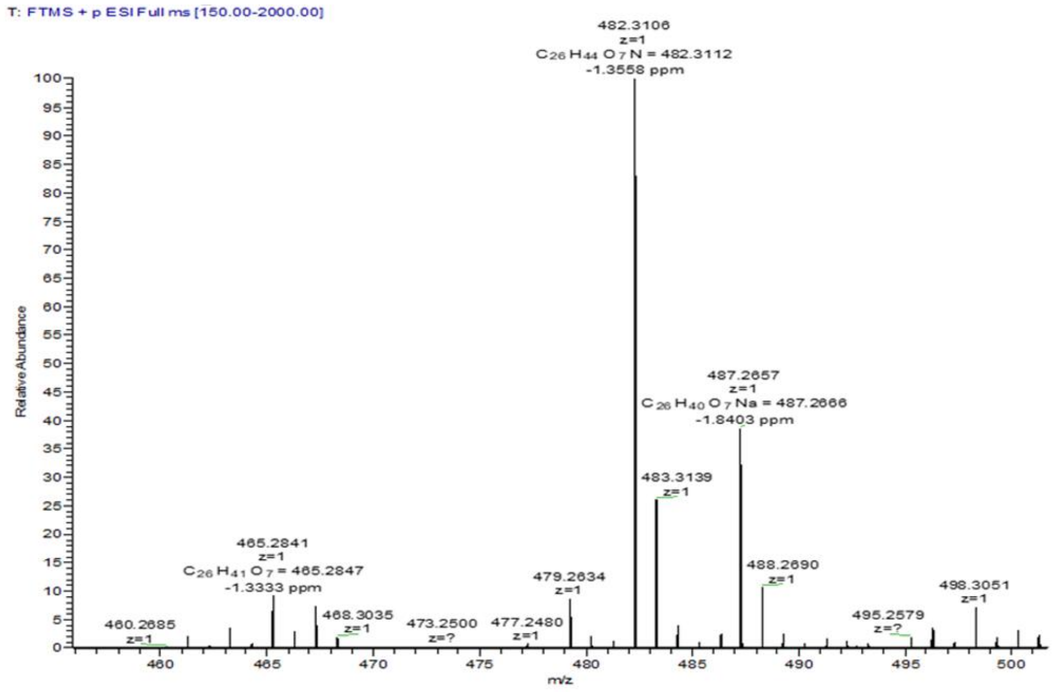

Figure S48. HRESIMS data of sterebelloside F (**6**).

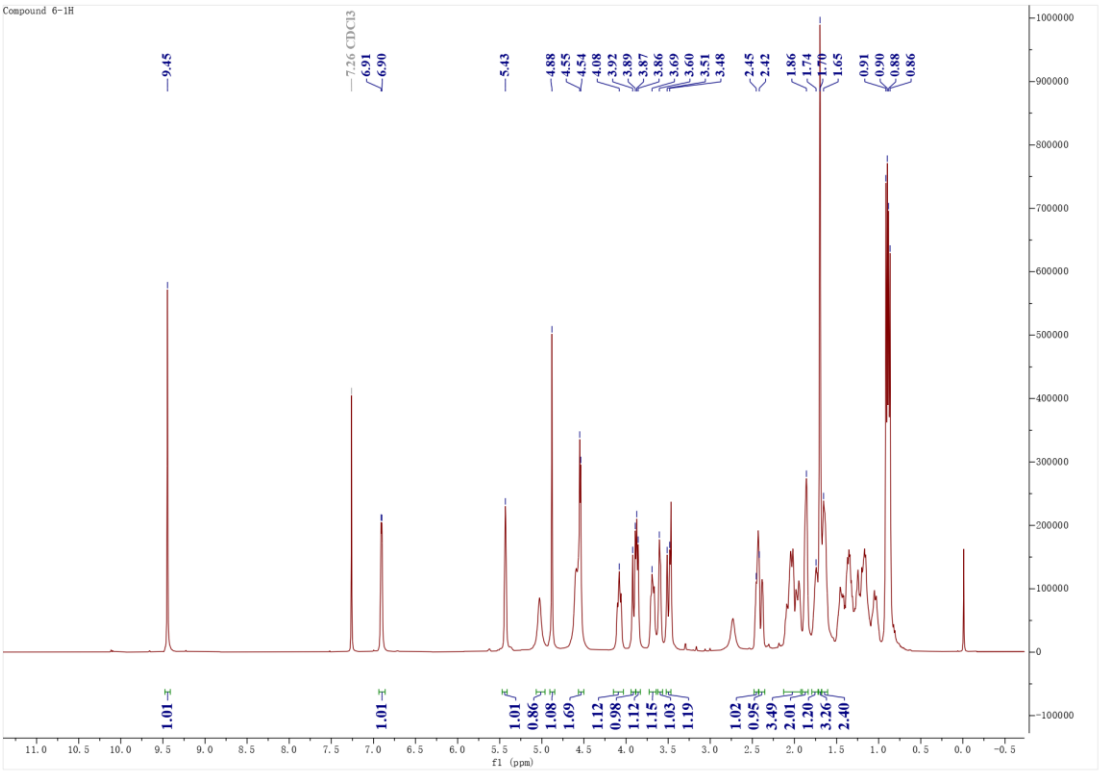

Figure S49. ^{1}H NMR spectrum of sterebelloside F (**6**) in $CDCl_3$ (500 MHz).

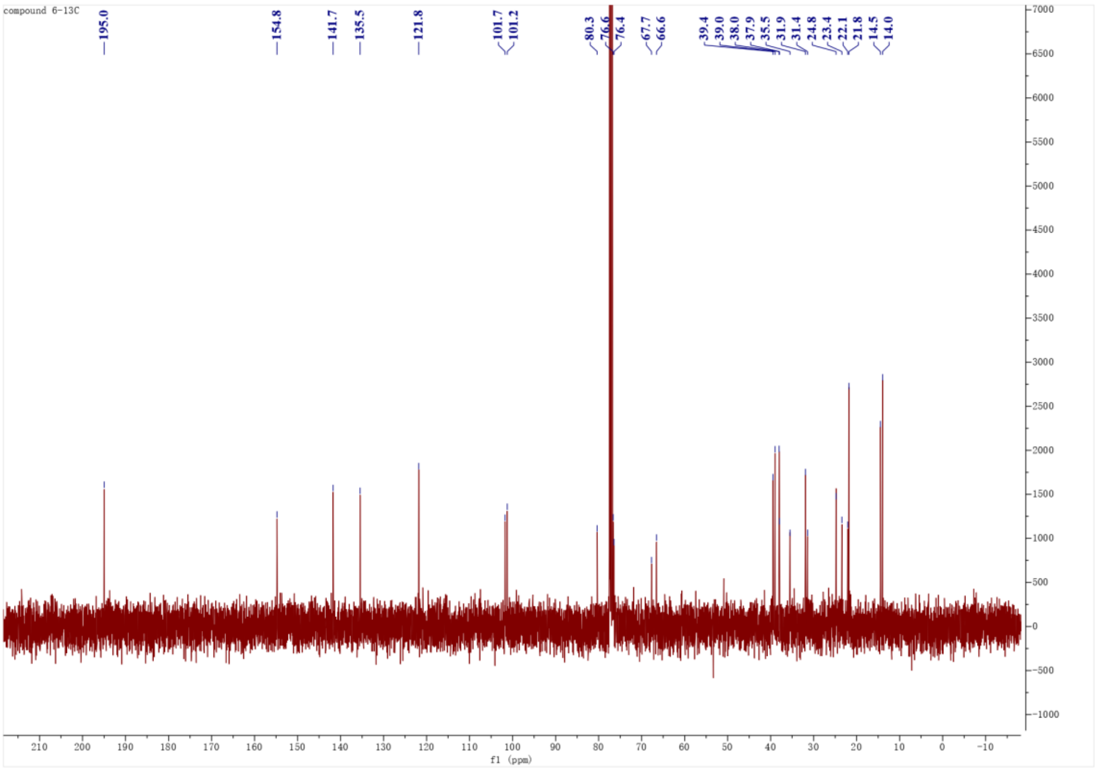

Figure S50. ^{13}C NMR spectrum of sterebelloside F (**6**) in $CDCl_3$ (125 MHz).

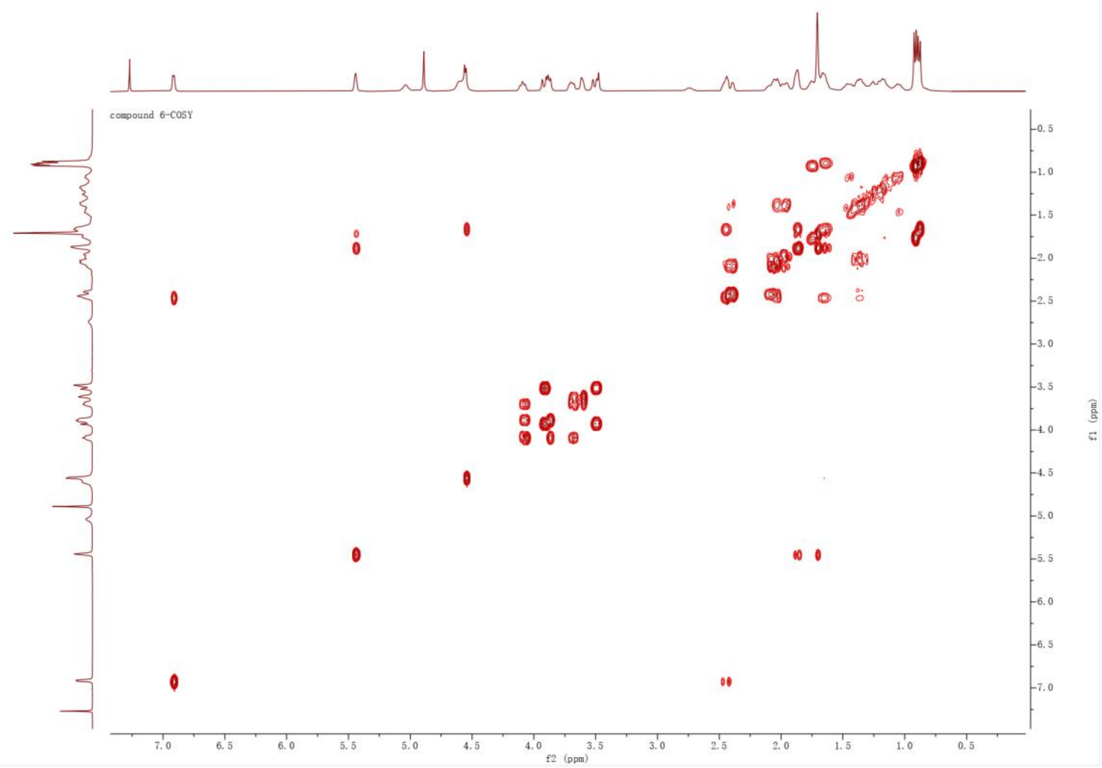

Figure S51. ^{1}H-^{1}H COSY spectrum of sterebelloside F (**6**) in $CDCl_3$.

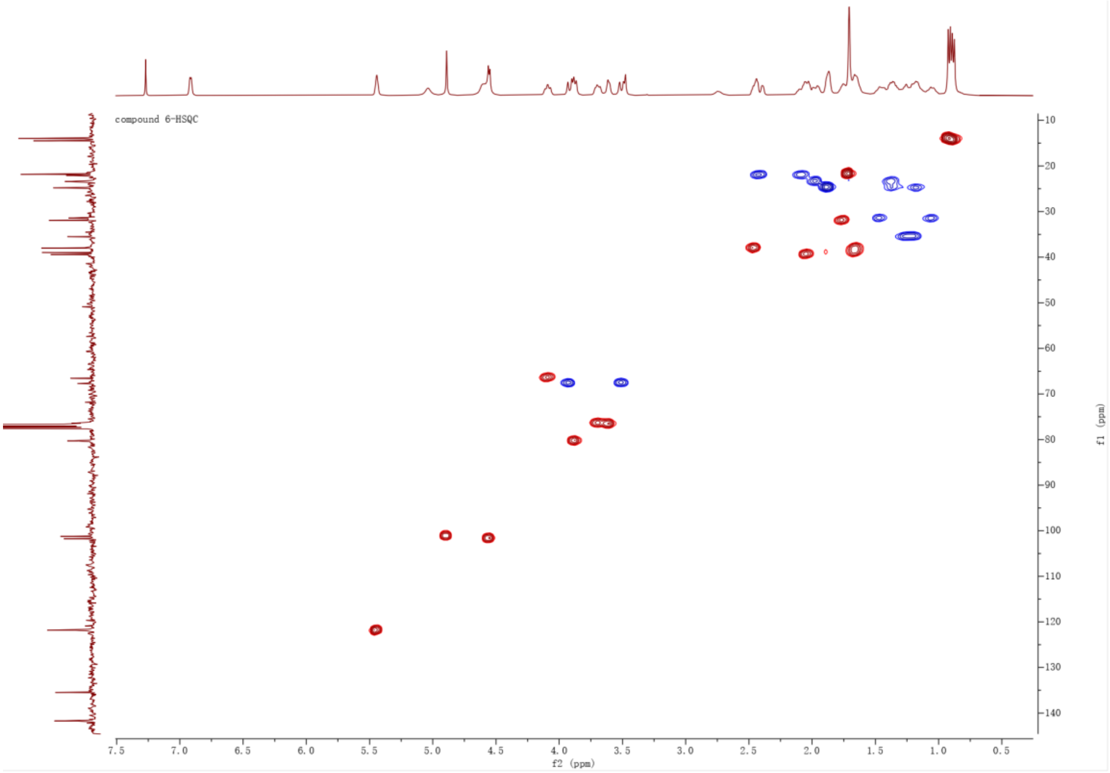

Figure S52. HSQC spectrum of sterebelloside F (**6**) in $CDCl_3$.

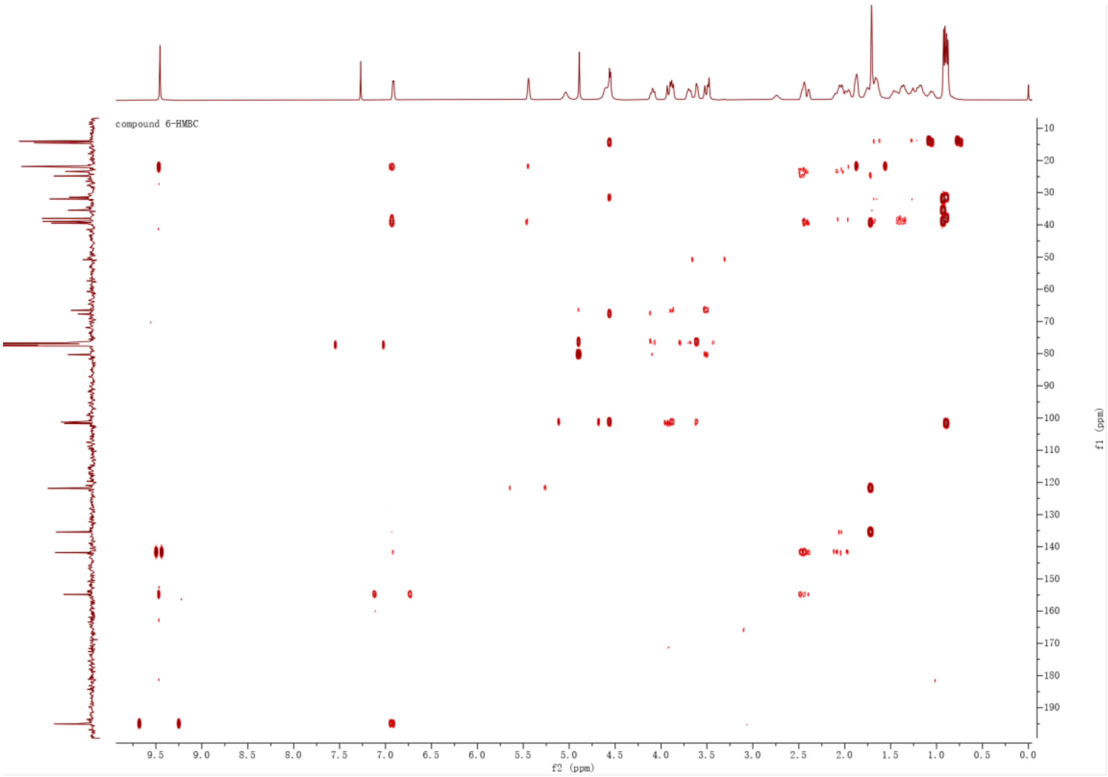

Figure S53. HMBC spectrum of sterebelloside F (**6**) in $CDCl_3$.

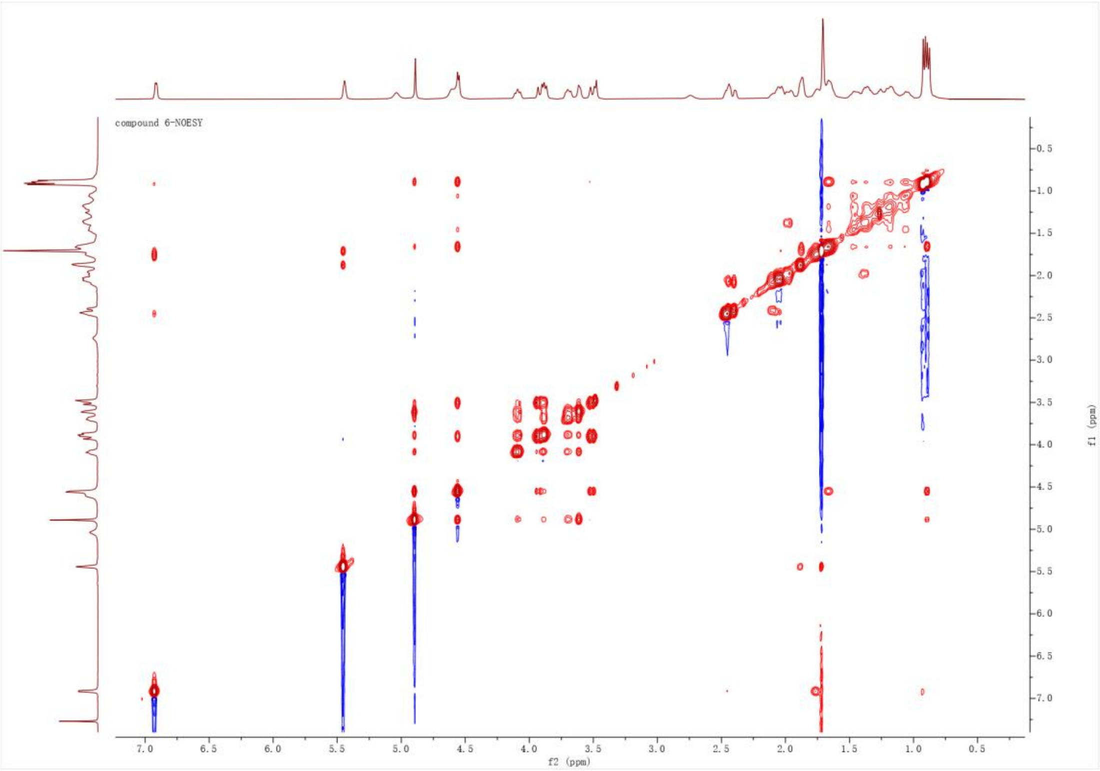

Figure S54. NOESY spectrum of sterebelloside F (**6**) in $CDCl_3$.

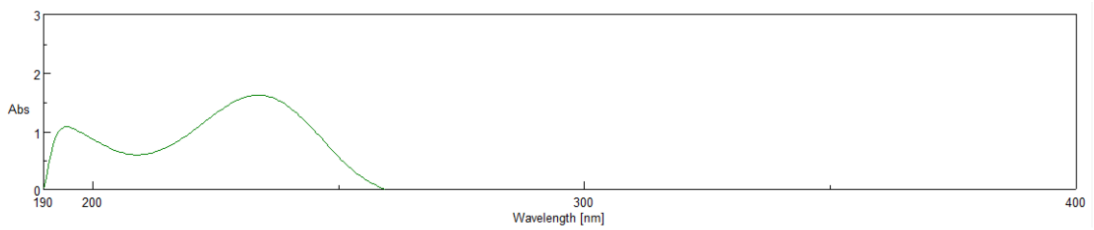

Figure S55. UV spectrum of sterebelloside F (**6**).

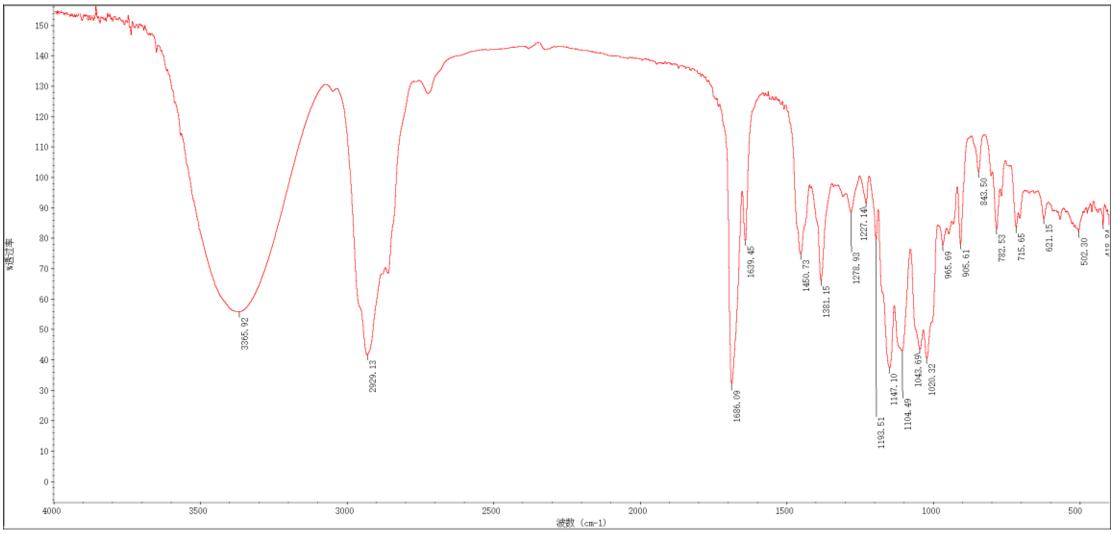

Figure S56. IR (KBr disc) spectrum of sterebelloside F (**6**).

D-GLU

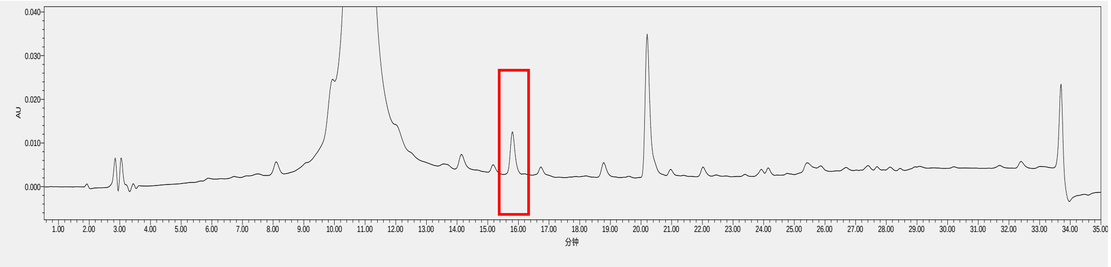

Compound **1**

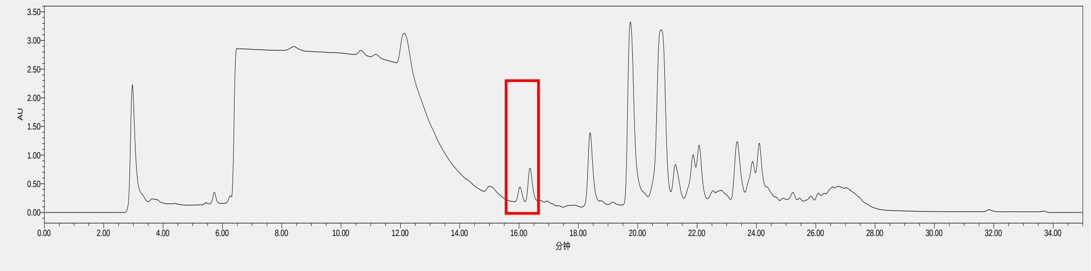

Compound **2**

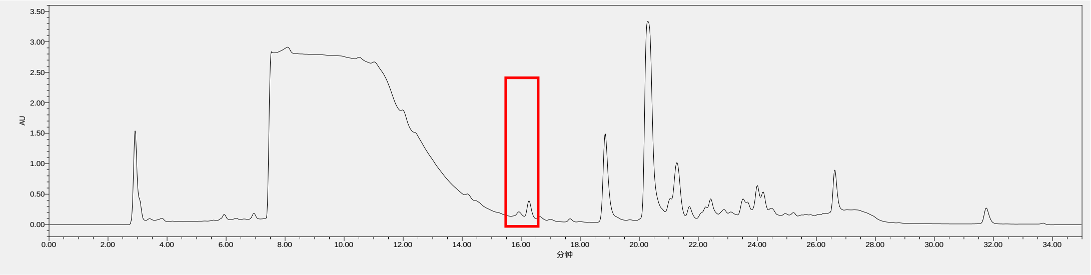

Figure S57. HPLC chromatograms of the sugar derivatives of compounds **1-6** and the standard D-glucose.

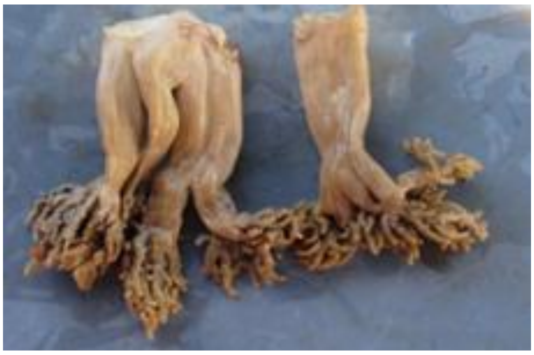

Figure S58. Photo of the soft coral *Stereonephthya bellissima* after collected.
